# Microneedle system for tissue engineering and regenerative medicine

**DOI:** 10.1002/EXP.20210170

**Published:** 2023-01-21

**Authors:** Yixin Zhang, Yanteng Xu, Huimin Kong, Jiabin Zhang, Hon Fai Chan, Jiasi Wang, Dan Shao, Yu Tao, Mingqiang Li

**Affiliations:** ^1^ Laboratory of Biomaterials and Translational Medicine Center for Nanomedicine The Third Affiliated Hospital Sun Yat‐sen University Guangzhou China; ^2^ Institute for Tissue Engineering and Regenerative Medicine School of Biomedical Science The Chinese University of Hong Kong Hong Kong China; ^3^ School of Biomedical Engineering Sun Yat‐sen University Shenzhen China; ^4^ Institutes of Life Sciences School of Medicine South China University of Technology Guangzhou China; ^5^ Guangdong Provincial Key Laboratory of Liver Disease Research Guangzhou China

**Keywords:** microneedle, regenerative medicine, tissue engineering

## Abstract

Global increasing demand for high life quality and length facilitates the development of tissue engineering and regenerative medicine, which apply multidisciplinary theories and techniques to achieve the structural reconstruction and functional recovery of disordered or damaged tissues and organs. However, the clinical performances of adopted drugs, materials, and powerful cells in the laboratory are inescapably limited by the currently available technologies. To tackle the problems, versatile microneedles are developed as the new platform for local delivery of diverse cargos with minimal invasion. The efficient delivery, as well as painless and convenient procedure endow microneedles with good patient compliance in clinic. In this review, we first categorize different microneedle systems and delivery models, and then summarize their applications in tissue engineering and regenerative medicine mainly involving maintenance and rehabilitation of damaged tissues and organs. In the end, we discuss the advantages, challenges, and prospects of microneedles in depth for future clinical translations.

## Graphical abstract

In this review, we have summarized the state‐of‐the‐art microneedles for tissue engineering and regenerative medicine, from their classifications and rational design, to the representative applications involving maintenance and rehabilitation of damaged tissues and organs, and the in‐depth insight into their current challenges and future directions.

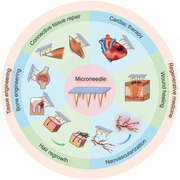

## 1 INTRODUCTION

Tissue and organ damage or dysfunction caused by trauma, disease, and aging are consistently great challenges in life sciences and health.^[^ ^1^ ^]^ Currently, clinical treatment usually employs protocols such as surgical repair, organ transplantation, artificial scaffolds implantation, and mechanical device intervention to enable patients to reconstruct, restore or compensate for the lost functions to varying degrees.^[^ ^2^, ^3^, ^4^, ^5^ ^]^ While these methods have benefited many patients, they have also revealed fatal weaknesses, such as surgical risks, limited donor availability, incompatibility with the human body, and insufficient function restoration. With advances in cell and molecular biology, engineering, and materials science, the late 1980s and early 1990s saw the birth of “tissue engineering,” which opened a whole new path for regenerative medicine.^[^ ^6^ ^]^ However, even to date, there is still a lack of practical techniques and methods for efficient targeting of diseased tissues, delivery of diverse therapeutic components, and precise regulation of the local microenvironment. Fortunately, the emerging microneedles offer a powerful solution to these problems.

Over the past 20 years, great progress has been made in the development of microneedle‐based drug delivery systems.^[^ ^7^ ^]^ Microneedle is considered as a promising platform for drug delivery because of its good safety, high efficiency, and painlessness.^[^ ^8^ ^]^ Generally, a microneedle patch is composed of an array of micron‐sized needles and a supporting base. Microneedles can penetrate the stratum corneum in a minimally invasive manner and create channels to transport small molecule drugs, large molecules (peptides, proteins, and nucleic acids), nanoparticles, and tissue fluids for precise treatment at the local sites.^[^ ^9^ ^]^

Typically, microneedles possess needles with 100–1000 µm in length and 1–10 µm in tip diameter.^[^ ^10^ ^]^ The configuration means that the maximum reaching depth is only within the distance of the epidermis and upper dermis, but does not cause irritation or damage to the nociceptive nerves and blood vessels underneath (1–2 mm in depth). The modulation of microneedle geometry can alter the mechanical strength and insertion depth, which determines the force required for microneedle inserting into the skin.^[^ ^11^ ^]^ Microneedles composed of soluble, insoluble, degradable, or non‐degradable materials can achieve rapid, slow, or stimulus‐responsive drug release, to meet different application cases.^[^ ^12^ ^]^ With increasing research, microneedles have already shown great potential for anti‐tumor therapies,^[^ ^13^, ^14^, ^15^, ^16^, ^17^ ^]^ hypoglycemic applications,^[^ ^18^, ^19^ ^]^ and vaccination usage.^[^ ^20^, ^21^ ^]^

**FIGURE 1:**
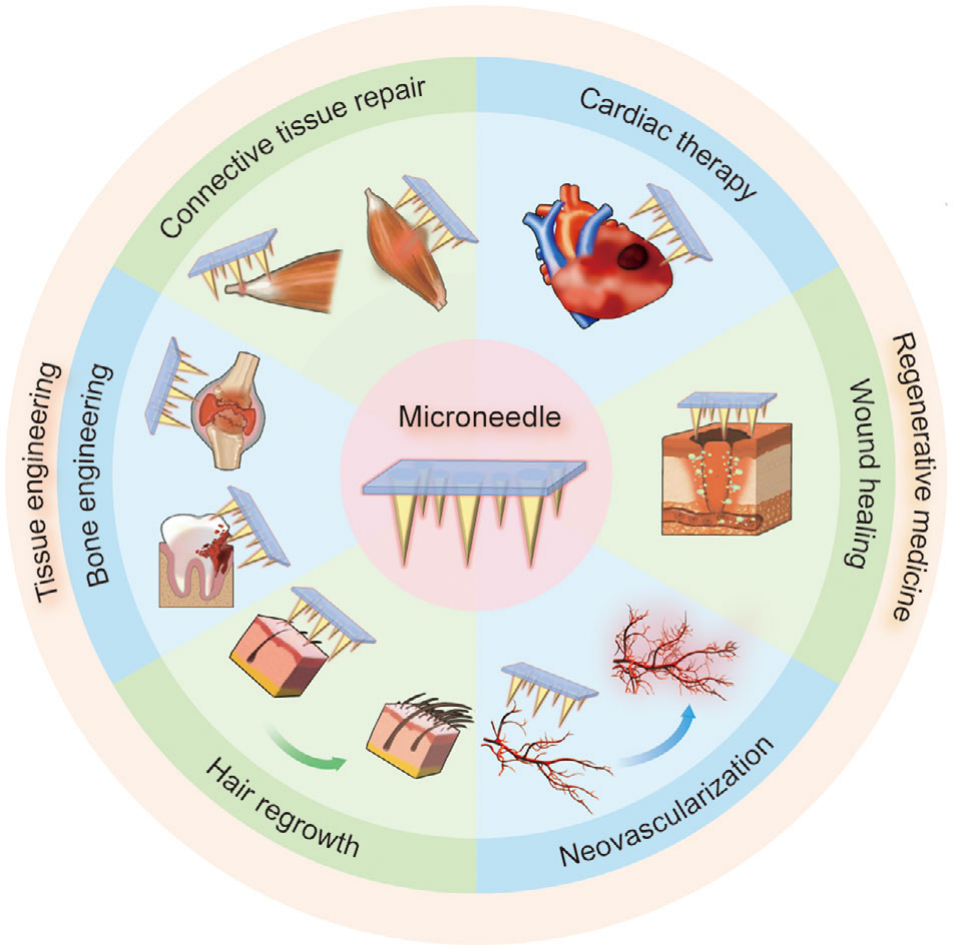
Schematic illustration of microneedles’ applications in tissue engineering and regenerative medicine

Promisingly, the advent of microneedles also spurs a wave in tissue engineering and regenerative medicine.^[^ ^22^, ^23^, ^24^ ^]^ The unique delivery method of microneedles allows for a wider application range in this aspect. They can not only work as the vehicles of cells or bioactive molecules regulating cell proliferation and differentiation, but also integrate with scaffold materials to maintain cellular activities. In this review, six categories of microneedles with different drug delivery mechanisms are introduced. Meanwhile, we also summarize the representative applications of various microneedles in tissue engineering and regenerative medicine, including repairing defective connective tissue and skeletal muscle, treating bone‐related and cardiac diseases, promoting neovascularization and wound healing, and fulfilling hair regrowth (Figure 1). In the end, challenges constraining the current development and outlooks for future research are discussed in depth.

## 2 CLASSIFICATION OF MICRONEEDLES

**FIGURE 2:**
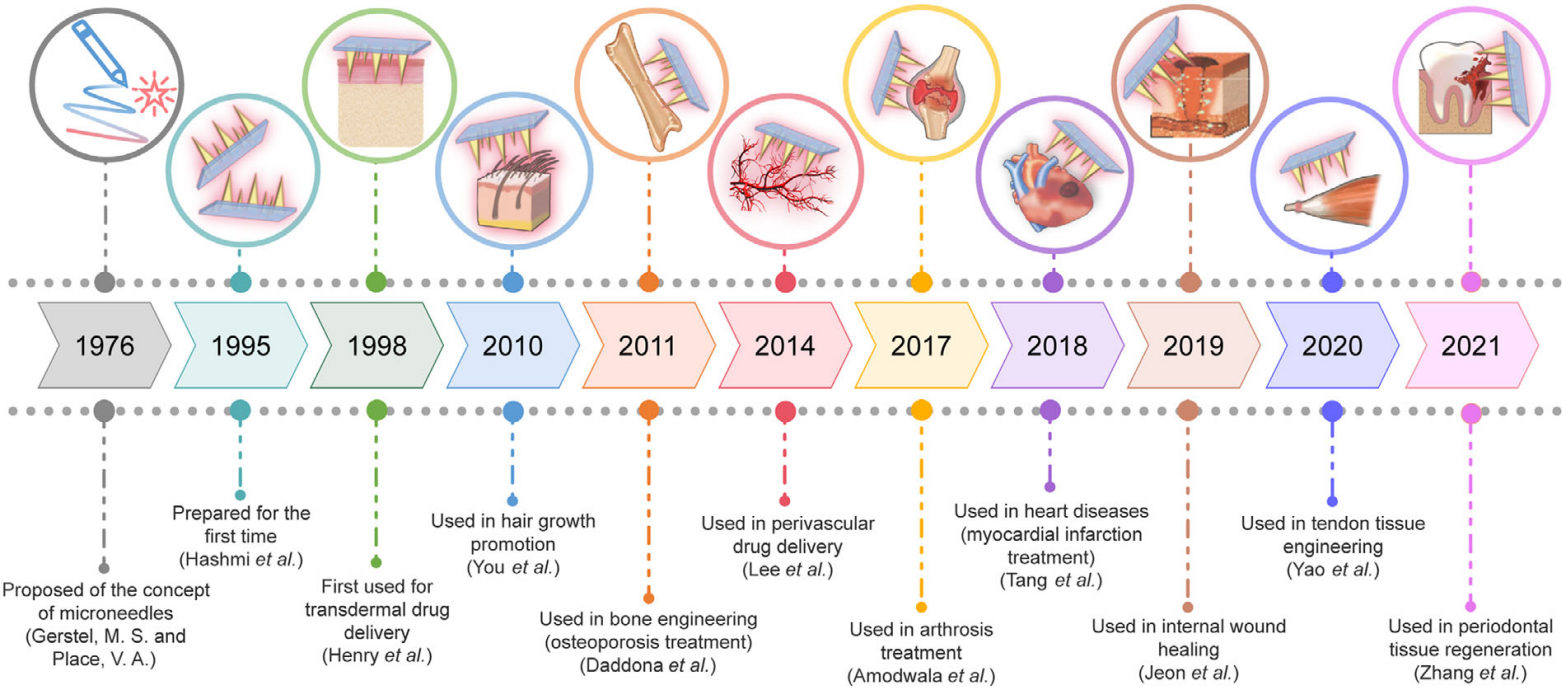
A brief timeline of the development and applications of microneedles in tissue engineering and regenerative medicine

Microneedle technology has been used in drug delivery since 1998, when Henry et al. first applied microneedles to the transdermal delivery of drugs.^[^ ^25^ ^]^ Figure 2 summarizes the timeline of microneedles’ development in the application of tissue engineering and regenerative medicine. Microneedle is a new and minimally invasive drug delivery platform that can break through the skin's stratum corneum barrier, creating multiple micro‐sized pores on the skin's surface and significantly increasing the efficiency of drug delivery. At the same time, advanced micro‐molding technology allows the fabrication of core–shell microneedles, double‐layered microneedles, and needle‐tip enrichment microneedles which have optimized drug release properties for better application in tissue engineering and regenerative medicine. By adjusting the size and shape, microneedles can be personalized to satisfy the needs of different patients.

**FIGURE 3:**
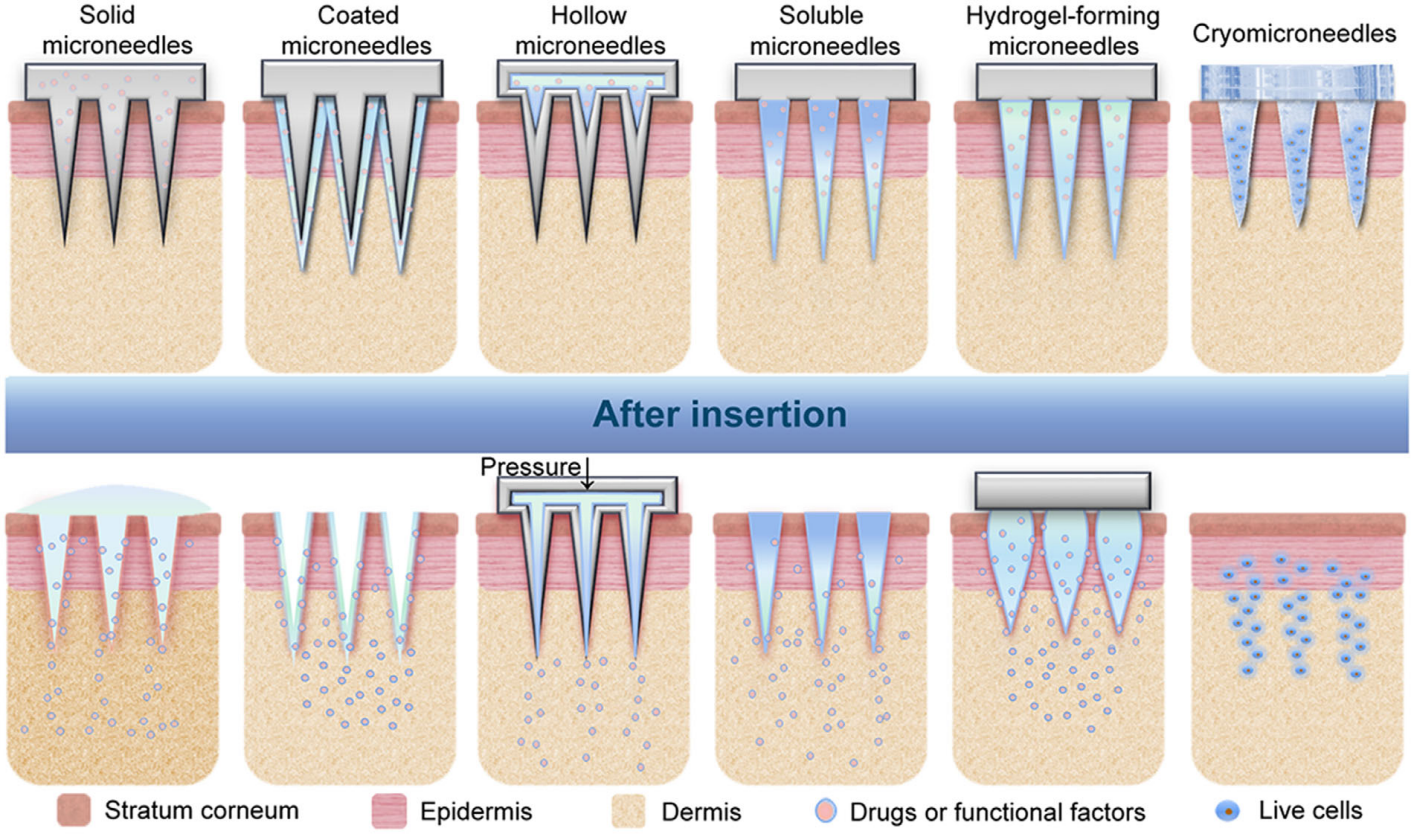
Types of microneedles and their corresponding drug delivery mechanisms

**FIGURE 4:**
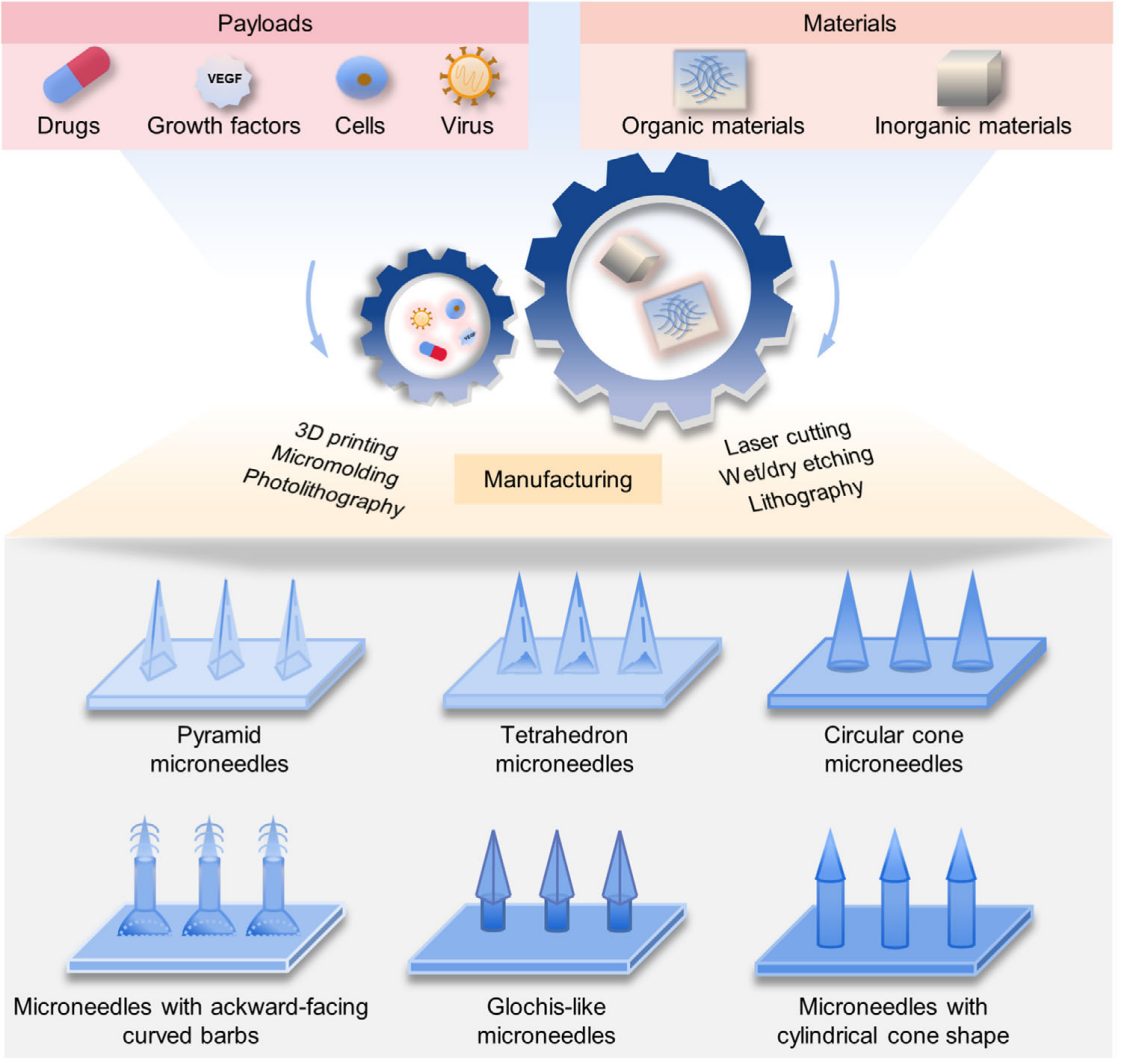
Microneedles can be prepared from a variety of materials and loaded with a wide range of payloads to perform different functions. Advanced manufacturing techniques enable diverse shapes of microneedles, which can increase drug loading and adhesion abilities

Currently, there are six main categories of microneedles according to the delivery modes: solid microneedles, coated microneedles, hollow microneedles, soluble microneedles, hydrogel‐forming microneedles, and cryomicroneedles.^[^ ^26^, ^27^ ^]^ Figures 3 and 4 illustrate the delivery mechanisms of these six types of microneedles and a variety of processing methods for the preparation of microneedles with diverse shapes and payloads, respectively.

### 2.1 Solid microneedles

Solid microneedles are usually prepared using metallic materials or non‐degradable polymers by laser cutting or mechanical/chemical etching.^[^ ^28^, ^29^ ^]^ For drug delivery, solid microneedles are first used to puncture the skin surface to form micro‐sized pores and the drug passively drains into the skin through the channels. Solid microneedles have good mechanical properties. However, once solid microneedle is broken during the drug delivery process, it will leave the non‐degradable needle tips in the skin, resulting in a hazard for the patient.

### 2.2 Coated microneedles

The materials used for fabricating the body of coated microneedles are similar to those for solid microneedles. The coating formulations require consideration of numerous factors including surfactants, thickening agents, and stabilizers. The main preparation methods of coatings include dip coating, drop coating, layer‐by‐layer coating, and spray coating.^[^ ^30^, ^31^ ^]^ Coated microneedles load drugs on the surface of the microneedle tips, which can be released under certain conditions. The drug delivery process is relatively simple, however the surface area of coated microneedles limits drug loading capacity.

### 2.3 Hollow microneedles

Hollow microneedles are mainly prepared using digitally controlled micromechanical systems, which is relatively time‐consuming and expensive.^[^ ^32^ ^]^ Hollow microneedles store drugs in either the central hollows of needle tips or the cavity of the supporting base. Employing an external drive, the drug is injected subcutaneously at the same time as the microneedle is pierced into the skin, enabling a rapid release of loaded cargos. Hollow microneedles allow the injection of various drugs with controllable dosages for different needs. However, due to the dense nature of the dermal tissue, the release of the drug is susceptible to blockage, which affects drug delivery.

### 2.4 Soluble microneedles

Soluble microneedles are mainly prepared from dissolvable and degradable polymers.^[^ ^33^ ^]^ Drugs are distributed in the needle tips and released in response to certain stimuli after piercing. They do not require the removal of the needle tips after formation of micro‐apertures. On the premise of meeting the required mechanical properties, choosing a highly biocompatible microneedle material can effectively improve application safety.

### 2.5 Hydrogel‐forming microneedles

Hydrogel‐forming microneedles are typically prepared from a polymeric matrix using the multiple‐step micro‐molding technique.^[^ ^34^ ^]^ This microneedle array rapidly absorbs tissue fluid upon insertion into the skin, causing the hydrogel to swollen and creating open pores within the gel network to release the loaded drugs.^[^ ^35^ ^]^ The drugs then penetrate and diffuse into the deeper tissues through the tissue fluid. The gelled microneedle can be removed intact after insertion into the skin. In addition, the drug release rate can be controlled by adjusting the crosslinking density of hydrogels.

### 2.6 Cryomicroneedles

Cryomicroneedles are initially designed to carry and store living mammal cells or bacteria inside the needles by scientists from the City University of Hong Kong.^[^ ^27^, ^36^ ^]^ During administration, the cryomicroneedles are punctured into the skin and dissolved, releasing the loaded cells or bacteria. This innovative technology allows the cell‐loaded microneedles to be stored for several months when cryopreservation in liquid nitrogen. One of the major clinical potentials of cryomicroneedles is intradermal delivery of dendritic cell (DC) vaccines.^[^ ^27^ ^]^ Cryomicroneedles solve the problems of time‐consuming and complex production procedures of conventional vaccination. Despite the high requirements of materials and production conditions, cryomicroneedles open up a whole new era of microneedle applications in cell therapy.

With the advances in materials and fabrication techniques, microneedles are in fast development, while they still show different advantages and disadvantages. Table 1 summarizes the fabrication methods and the main materials used for each category of microneedles, as well as their advantages and limitations.

**TABLE 1:** Summary of six categories of microneedles

Category	Materials	Fabrication	Advantages	Limitations	Ref.
Solid microneedles	Metals, ceramics, silicon, non‐degradable polymers	Laser cutting and electrodeposition, ceramic micromolding and sintering; etching, photolithography, and lithography	Earliest to be developed Good mechanical performance Well‐developed preparation technologies	Short duration of action and inability to accurately control the dose administered due to dynamic self‐healing process of micro‐channels in the skin Brittle	^[^ ^30^, ^144^, ^145^ ^]^
Coated microneedles	Body materials: similar to solid microneedles Coating formulations: Surfactants (Lutrol F68 tween 20, poloxamer 188, and Quil‐A), thickening agents (methylcellulose, carboxymethylcellulose sodium salt, hyaluronic acid, sodium alginate), stabilizers (trehalose and sucrose) Compounds coated: small molecules, macromolecules (peptide, proteins), vaccines, nanomaterials, nucleic acids (DNA, siRNA)	Compounds coated: small molecules, macromolecules (peptide, proteins), vaccines, nanomaterials, nucleic acids (DNA, siRNA)	Reusable Stable coating Suitable for the delivery of a wide range of substances including small and large molecules	Low drug loading capacity Coatings affect the mechanical properties of the needle tip Drug retention caused by friction force	^[^ ^144^, ^146^, ^147^, ^148^, ^149^, ^150^, ^151^, ^152^, ^153^, ^154^, ^155^, ^156^, ^157^, ^158^, ^159^ ^]^
Hollow microneedles	Silicon, silica capillaries, NanoFil needles, stainless steel, glass, hollow microstructured transdermal system (hMTS), MicronJet600 microneedles	Microelectromechanical systems (MEMS) techniques such as laser cutting and laser ablation, wet etching and deep reactive‐ion etching, 3D printing technology, drawing lithography	Maximum drug loading capacity due to the hollow structure Controlled dose and rate of administration	Sophisticated and complex preparation process Needle breakage caused by improper force during administration Vulnerability to the drug release rate	^[^ ^32^, ^160^, ^161^, ^162^, ^163^, ^164^, ^165^, ^166^, ^167^, ^168^, ^169^, ^170^, ^171^ ^]^
Soluble microneedles	Dissolvable polymers including hyaluronic acid (HA), sodium alginate, dextran, gelatin, polyvinylpyrrolidone (PVP), carboxymethylcellulose, hydroxypropyl cellulose, polyvinyl alcohol (PVA) Degradable polymers including polylactic acid (PLA), polyglycolic acid (PGA), poly(lactic‐*co*‐glycolic acid) (PLGA), and chitosan (CS)	Micromolding, 3D printing technology, drawing technique, droplet‐born air blowing	Lower production costs Maintain the activity and stability of the drug Quantitative and controlled release of drugs	High requirements for the materials used Insufficient mechanical strength required to puncture skin	^[^ ^33^, ^170^, ^172^, ^173^, ^174^, ^175^ ^]^
Hydrogel‐forming microneedles	Poly(methylvinylether maleic anhydride) (PMVE/MA), PVA/CS, Phenylboronic acid (PBA), methacrylate‐based gelatin (GelMA)/PVA, GelMA/PLGA, N‐isopropylacrylamide (NIPAM)/silk fibroin	Micro‐molding method	Simple preparation process and easy to scale up production Resist the closure of skin micropores Unbreakable and easily removed intact	Relatedly poor needle shape and insufficient mechanical strength Easily detached Increasing wound infection risk due to the excellent biocompatibility	^[^ ^117^, ^123^, ^127^, ^176^, ^177^, ^178^, ^179^, ^180^, ^181^ ^]^
Cryomicroneedles	Cryoprotectants (dimethyl sulfoxide) incorporating with sucrose (a non‐penetrating cryoprotectant)	Micro‐molding method	Deliver living cells Improve the utility of DC vaccination Package other types of therapeutic cells Facilitate the development of cell therapy against cancer and other diseases	Must be used immediately after removal from low temperature Production conditions: whether cryo‐sterilization can meet sterility standards Limited cell loading capacity	^[^ ^27^, ^36^ ^]^

## 3 APPLICATION OF MICRONEEDLES IN TISSUE ENGINEERING AND REGENERATIVE MEDICINE

Damage and defects in human tissues can lead to functional impairment. The traditional therapeutic method is autologous tissue transplantation. This approach can achieve satisfactory results to some extent, but it's likely to cause some complications and additional damage. Organ failure in the human body can be partially saved by pharmacological treatment and allotransplantation with the problems of gastrointestinal side effects, low compliance, extremely high costs, and limited organ donors. Since the 1980s, the treatment of patients with tissue defects and organ failure has been brought to light with the introduction of tissue engineering.^[^ ^37^ ^]^ Tissue engineering and regenerative medicine combine multidisciplinary theories and technologies to develop therapies based on tissue and organ transplantation, and promote endogenous recovery and regeneration. It aims to restore the normal functions of injured tissues or organs by studying the features and mechanisms of tissue repair and regeneration in vivo. In addition to the widely used drugs and growth factors, various natural compounds, such as chitosan, tannic acid, silk fibroin, cellulose, and synthetic polymers have also been evidenced with therapeutic functions in tissue regeneration.^[^ ^38^, ^39^, ^40^ ^]^ The appearance of microneedles allows them to achieve target therapeutic effects through transdermal drug delivery. Stem cells, including embryonic stem cells, mesenchymal stem cells (MSCs), and induced pluripotent stem cells (iPSCs), are also powerful tools in regenerative medicine because they can differentiate into desired cells in response to different stimuli, secrete functional factors, and regulate immunity.^[^ ^41^, ^42^, ^43^ ^]^ Although the use of stem cells requires strict conditions, a growing number of studies have shown successful delivery of stem cells to injured tissues and functional restoration in a precise and safe manner via microneedles. This section provides an overview of using microneedles in tissue engineering and regenerative medicine and discusses their potential applications.

### 3.1 Repairing defective connective tissues

Connective tissues are widely distributed in the human body and serve vital functions of connection, support, and protection. Tendon, as one of the typical connective tissues, joins muscle to bone implementing musculoskeletal system functions.^[^ ^44^ ^]^ According to the research statistics, about 30% of clinical consultation on musculoskeletal system diseases is related to tendon injuries.^[^ ^45^ ^]^ Overuse, aging, injury, or disease can bring defects to the tendon. Due to the lack of cells and blood vessels, the tendon's healing and reconstruction ability is poor and inadequate.^[^ ^46^ ^]^ Given the high incidence of tendon injuries and the lack of comprehensive repair solutions, tendon healing faces a huge clinical challenge and there is an urgent need for establishing new treatments.

The mammalian target of rapamycin (mTOR) signaling pathway is a significant regulator of cell biological processes including cell growth and metabolism.^[^ ^47^ ^]^ It has been confirmed that tendon's self‐healing ability can be highly improved by activating the mTOR signaling.^[^ ^46^ ^]^ Based on this, Yao et al. developed hydrogel microneedles with PVA needles containing parathyroid hormone (PTH).^[^ ^48^ ^]^ PTH is a systemic hormone exhibiting a positive impact on tendon bone healing process.^[^ ^49^, ^50^ ^]^ The PVA‐based hydrogel microneedles were fabricated using the freezing–thawing method,^[^ ^51^ ^]^ keeping PTH from denaturing. The PTH‐loaded microneedles were directly pressed into the rat skin to achieve transdermal transport of PTH. With the sustained release of PTH, mTOR signaling pathway could be activated. Moreover, the expression amounts of tendon‐related proteins, such as collagenase type 1 (Col1a) and decorin (DCN) were increased to promote efficient tendon repair with minimal infection.

**FIGURE 5:**
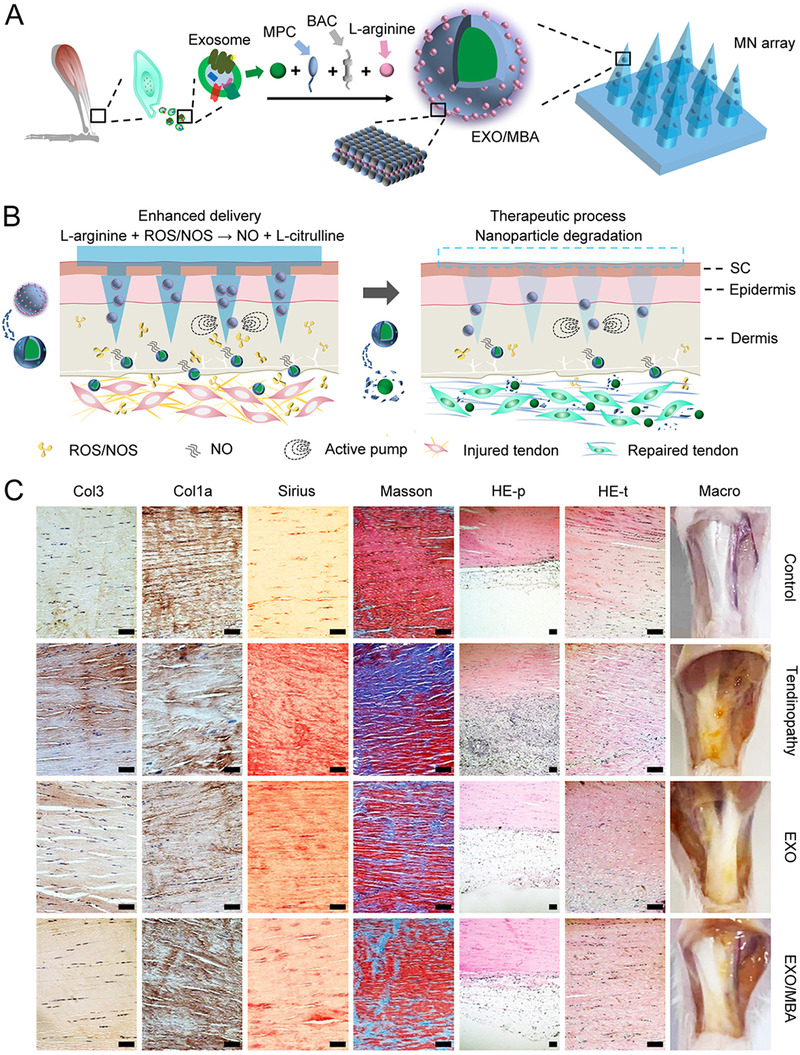
NO nanomotor‐driven EXO/MBA‐loaded microneedles for tendon healing. (A) Design of the microneedles array. (B) The healing process after application of EXO/MBA‐loaded MN array. (C) Evaluation of the repair effect of the EXO/MBA‐loaded MN array using immunohistochemistry of Col1a and Col3, Sirius staining, Masson staining, H&E staining of peritendineum (HE‐p), H&E staining of tendon (HE‐t) and Macro‐picture in control, tendinopathy, EXO, and EXO/MBA groups respectively (scale bar: 100 µm). Reproduced with permission.^[^ ^53^ ^]^ Copyright 2021, American Chemical Society

Due to the unique transdermal delivery property of microneedles, some drugs or substances with insufficient penetration, transitory persistence, and imprecise targeting can be used to treat tendon injuries. It has been shown that exosomes (EXOs) secreted by stem cells can promote the tissue repair and regeneration process.^[^ ^52^ ^]^ In a latest study, researchers used microneedles to deliver EXOs‐loaded 2‐methacryloyloxyethyl phosphorylcholine (MPC) and *N*,*N*′‐bis(acryloyl)cystamine particles (EXO/MBA) to treat Achilles tendinopathy.^[^ ^53^ ^]^ The shape of the microneedles is glochis‐like to keep strong adhesion at the joints, and a “smart” nitric oxide (NO) nanomotor was designed to provide the driving force for the EXOs to achieve the long‐term and stable release of EXOs (Figure 5A). When EXO/MBA entered the inflammatory microenvironment of Achilles tendinopathy, the L‐arginine on its surface was broken down into L‐citrulline and NO. This process was catalyzed by nitric oxide synthase or reactive oxygen species (ROS). The generated NO allowed EXOs to move deeper and exert anti‐inflammatory and repairing effects at the tendon injury site (Figure 5B). After treatment, the expression of Col1a increased significantly, and this is accompanied with the reduction of inflammation areas and rearrangement of collagen fibers (Figure 5C). Altogether, the microneedles loading NO nanomotor‐driven EXOs had obvious advantages over traditional tendon therapies and provide an idea for the treatment of movement system diseases.

In the above studies, although the microneedles were fabricated in various forms and different drugs were loaded, all of them achieved excellent therapeutic goals. Overall, the applications of diverse microneedles offer a new drug delivery strategy for clinical tendon repair. At the same time, microneedles can also be used in the research of other connective tissue diseases.

### 3.2 Repairing injured skeletal muscle

Skeletal muscle is an important tissue that is widely present in the human body and is responsible for movement.^[^ ^54^ ^]^ Skeletal muscle injuries accompanied by muscle fiber tears, inflammation, hemorrhage, and edema can lead to associated function loss and intolerable pain.^[^ ^55^ ^]^ Therefore, the treatment of skeletal muscle injuries becomes particularly important. Moxibustion is a Chinese traditional thermal treatment method in the clinic, but massive smoke from combustion and local high temperature make it urgent to develop a smoke‐free and more secure treatment form. Zhang et al. designed PVA‐based soluble microneedles containing carbonized wormwood (a photothermal agent) and prostaglandin E2 (PGE2) to achieve a synergistic effect of photothermal therapy and drug treatment.^[^ ^56^ ^]^ Under 808 nm near‐infrared (NIR) irradiation, the temperature of microneedles raised quickly and steadily, exerting thermal therapeutic effect and accelerating drug release. Surgical muscle damage experiments showed more than 90% of muscle strength recovery in PGE2 + NIR group. Moreover, the number and area of muscle fibers increased substantially. The expression of specific stem cell markers was detected and results confirmed that the carbonized wormwood modified microneedles could induce muscle stem cells to proliferate and differentiate. Altogether, the photothermal microneedles create an anti‐burn and smoke‐free treatment based on moxibustion and bring benefits to the patients with skeletal muscle injuries in clinic.

The applications of microneedles in skeletal muscle‐related disease treatments can reduce side effects associated with traditional drug delivery methods and improve the therapeutic effect. With more in‐depth studies, a variety of new microneedles have been strengthened by advanced technologies including electrostimulation^[^ ^57^, ^58^ ^]^ and magnetic response^[^ ^59^ ^]^ verified by controllably delivering insulin in the intestine, which brings hopes for repairing other types of skeletal muscle injuries.

### 3.3 Treating bone‐related diseases

Bone‐related diseases are often caused by trauma, inflammation, infection, tumors, and osteoporosis.^[^ ^60^, ^61^ ^]^ In current drug injection treatments of bone defects, intravenous administration often fails to achieve effective drug concentration at the lesion site.^[^ ^62^ ^]^ Conventional transplantation methods not only increase patients' pain, but are also accompanied by the potential risk of infection and complications.^[^ ^63^ ^]^ Therefore, how to improve the therapeutic effect and patient compliance is a challenge needing to be overcome in orthopedic clinic. Xu and his team designed conductive microneedles as an anesthetic reservoir for transporting drugs to the nerve‐located bone tissues during dental surgery.^[^ ^64^ ^]^ The microneedles were prepared using HA and (poly(3,4‐ethylenedioxythiophene) polystyrene sulfonate (PEDOT:PSS). PEDOT:PSS had excellent biocompatibility and conductivity.^[^ ^65^ ^]^ HA/PEDOT:PSS microneedles could reduce the electrical resistance of the skin. With the help of iontophoresis, lidocaine could be used for rapid anesthesia from the surface of the oral mucosa into the bone tissues.^[^ ^66^ ^]^ Although their study only reported the drug delivery to dental bone tissues, the conductive microneedles using iontophoresis technology have the potential to treat other orthopedic diseases, such as spine injury, with corresponding drug reservoir. More recently, Zhang et al. used microneedles to deliver antibiotics and cytokines to treat periodontitis, achieving immunomodulation and regeneration of periodontal tissue.^[^ ^24^ ^]^ This also illustrates the application potential of microneedles in intraoral drug delivery and provides innovative ideas and methods for periodontal tissue engineering.

Rheumatoid arthritis (RA) is one of the joint lesions and an autoimmune disease caused by several factors including exogenous (hostile environment and virus invasion) and endogenous (cellular, genic, and neural problems) factors.^[^ ^67^, ^68^ ^]^ To date, RA is usually treated by oral or injectable drugs including non‐steroidal anti‐inflammatory agents, corticosteroids, and disease‐modifying anti‐rheumatic drugs.^[^ ^69^ ^]^ However, due to the side effects such as poor patient compliance, gastrointestinal disturbance, and other toxicity risks associated with these administrations, there is an urgent need to develop a new form of drug delivery.^[^ ^70^ ^]^ With this demand, transdermal drug delivery systems, represented by microneedles, are becoming popular in the treatments of RA. Recently, a melittin‐loaded polymeric microneedle patch was developed by Du et al.^[^ ^71^ ^]^ Methacrylate groups were used to modify HA to obtain MeHA with better‐sustained drug release and drug protection properties.^[^ ^72^ ^]^ In the Adjuvant‐Induced Arthritis (AIA) rodent model, the melittin‐loaded MeHA microneedles could inhibit further swelling of the rat's paw. Meanwhile, the rise of regulatory CD4^+^ T cells was accompanied by a fall of TNF‐α and IL‐17, which demonstrated an inhibitory effect on RA and a protective effect on articular cartilage. The transdermal drug delivery performed by microneedles reduced inflammation, protected joint integrity, and had fewer side effects and toxicity, which might be an alternative strategy for the treatment of RA in the future.

**FIGURE 6:**
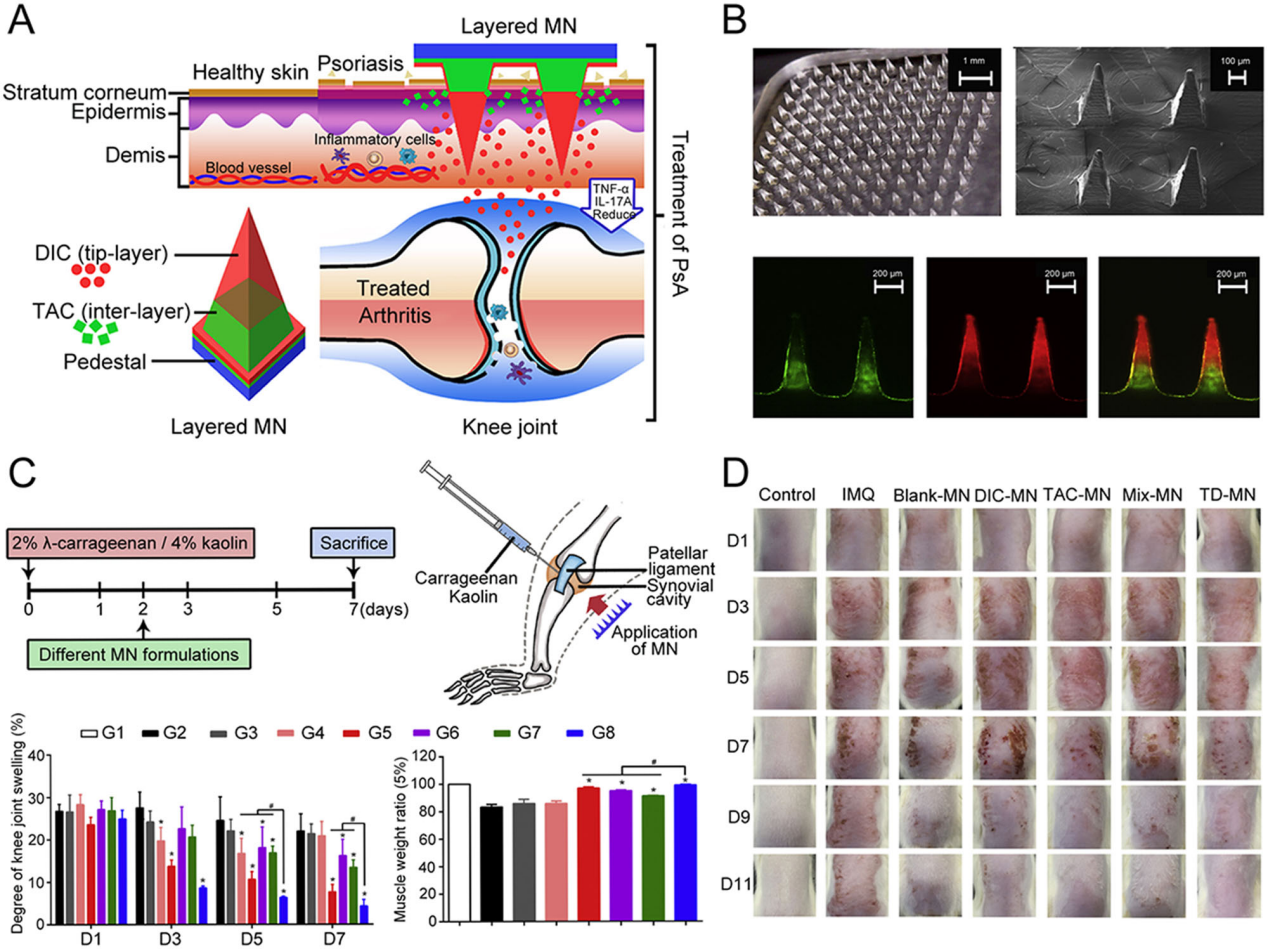
Multilayered microneedles for both PsA and skin psoriatic lesion treatment. (A) Design of multilayered dissolving microneedle system loading TAC and DIC. (B) Fabrication and characterization of multilayered microneedles. (C) Arthritis model establishment and treatment. G1: healthy group, G2: RA model group, G3: Blank‐MN, G4: DIC‐injection, G5: DIC‐MN, G6: TAC‐MN, G7: MIX‐MN, G8: TD‐MN. Significant differences were calculated using ANOVA test. ^∗^ *p* < 0.05 in comparison with RA model group; ^#^ *p* < 0.05 in comparison with TD‐MN. (D) Images of psoriatic skin lesions treated with different MN formulations. Reproduced with permission under a Creative Commons CC BY‐NC License.^[^ ^76^ ^]^ Copyright 2021, Elsevier

Psoriatic arthritis (PsA) is a chronic joint disease.^[^ ^73^ ^]^ Symptoms are complex and often occur accompanied by skin psoriatic lesions.^[^ ^74^ ^]^ The patients' epidermis is often abnormally thickened, which substantially limits the penetration and efficacy of topical medications.^[^ ^75^ ^]^ Similar to RA, oral, local injection, and systemic administration for PsA have certain side effects and cannot treat diseased skin at the same time as joints. On this basis, Xu and his team designed a multilayered microneedle (MN) called TD‐MN, which allowed different drugs to be administered at different sites (Figure 6A).^[^ ^76^ ^]^ The tip layer of the microneedle carried anti‐inflammatory diclofenac (DIC), while the inter layer contained immunosuppressant tacrolimus (TAC) and the pedestal consisted of HA, Dextran, and PVP (Figure 6B). After the microneedles penetrated skin, DIC at the tip layer permeated deeper into the inflamed joints (50–300 µm in depth beneath the epidermis surface) and this release process was continuous with a long residence time. The inter layer of the microneedle stayed only in the epidermis (0–100 µm in depth), releasing TAC to treat the skin psoriatic lesions. The therapeutic effect of TD‐MN on PsA was evaluated by a RA model. A reduction of ∼ 4.33% in joint swelling and prevention of muscle atrophy were observed (Figure 6C). In the skin psoriasis model, the skin barrier was restored by relieving erythema and hemorrhage after TD‐MN treatment (Figure 6D). Overall, this layered microneedle system enables site‐specific drug delivery and is expected to meet the requirements of PsA treatment.

To sum up, although there have been effective drugs in the clinical treatment for some bone‐related diseases, the current administrations are usually associated with unavoidable side effects. Evolving microneedle technologies bring novel ways of delivering drugs to diseased bone tissues with painless, efficient local drug delivery, and high patient compliance, thereby holding great promise for further development.

### 3.4 Treating cardiac diseases

Cardiovascular disorders like myocardial infarction (MI) and ischemic myocardial disease, have become an intractable medical issue because of their high incidence and low curative rate.^[^ ^77^ ^]^ The effective treatments to re‐establish the function of infarcted myocardial tissue include reperfusion therapy,^[^ ^78^ ^]^ thrombolytic therapy,^[^ ^79^ ^]^ and cellular therapy.^[^ ^80^ ^]^ To date, stem cell‐based therapies are already applied for cardiac therapy and gained attention for their ability to promote injured heart regeneration. However, due to the low differentiation efficiency and retention rate of stem cells in a complex cardiac physiological environment, their direct administration increases the uncertainty and risk.^[^ ^81^ ^]^ One method to tackle this problem is to use microneedles, which can accelerate cell integration and retention through precision therapy.

**FIGURE 7:**
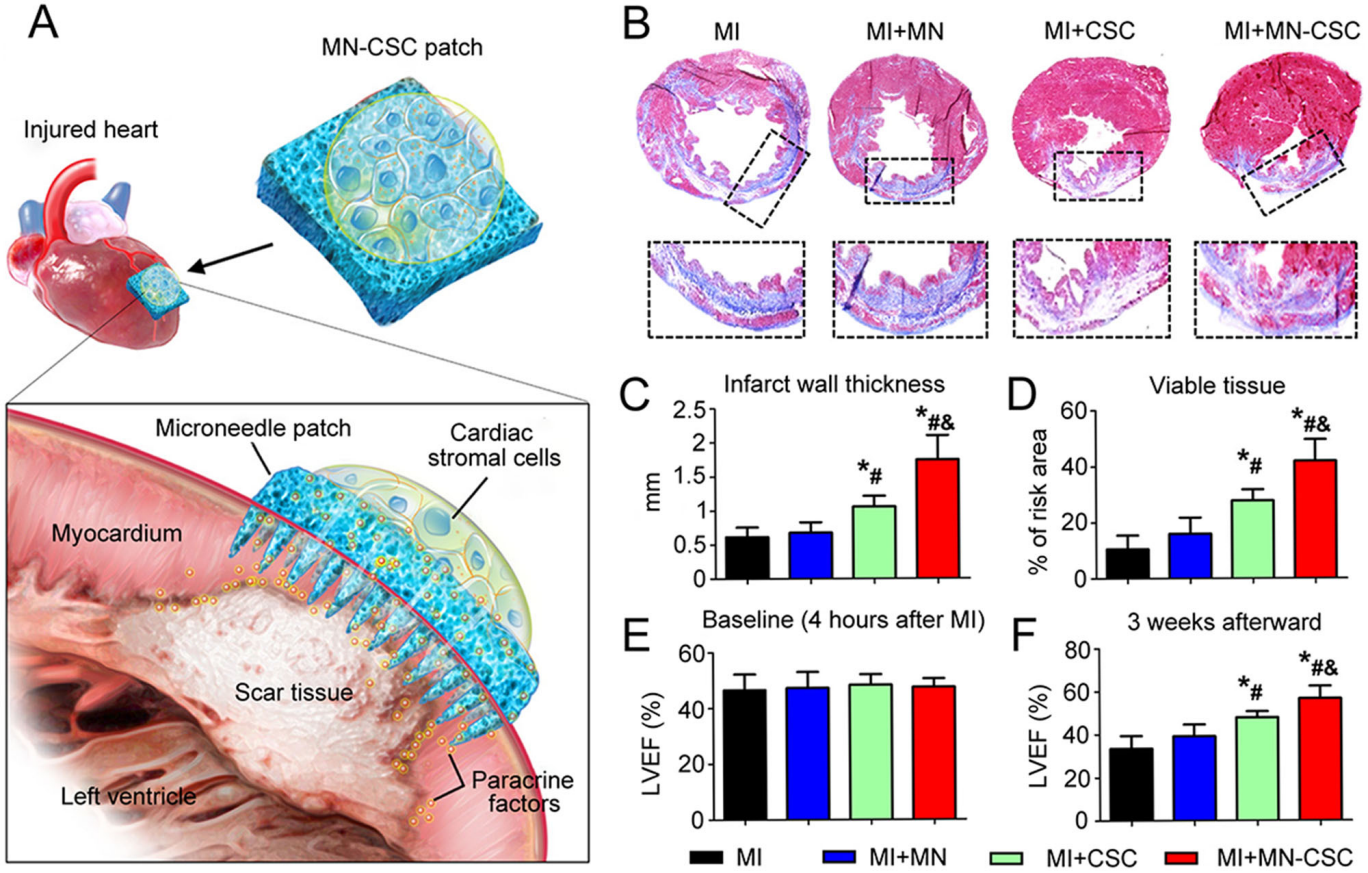
Heart‐derived CSCs‐loaded PVA microneedles for treating MI. (A) Illustration of the overall design of MN‐CSCs. (B) Masson's trichrome staining of heart morphology and fibrosis 3 weeks after applied with different conditions (red: viable tissue; blue: scar tissue). (C,D) Quantitative analyses of infarct wall thickness and viable tissue in the risk area based on Masson's trichrome staining. (E,F) LVEFs measured by echocardiography at 4 h (baseline) and 3 weeks (endpoint) after MI. ^*^ *p* < 0.05 when compared with the MI group; ^#^ *p* < 0.05 when compared with the MI + MN group; ^&^ *p* < 0.05 when compared with the MI + CSC group. Reproduced with permission under a Creative Commons CC BY‐NC License.^[^ ^84^ ^]^ Copyright 2018, American Association for the Advancement of Science

Heart‐derived cardiac stem/stromal cells (CSCs) in several clinical trials have been confirmed with positive therapeutic effects on patients suffering from MI.^[^ ^82^, ^83^ ^]^ Informed by this, Tang et al. developed PVA microneedles integrated with CSCs (MN‐CSCs) for MI therapy (Figure 7A).^[^ ^84^ ^]^ The MN‐CSCs were well biocompatible with neonatal rat cardiomyocytes (CMs) and could act as a communication and transportation channel between CSCs and the injured myocardium. CSCs in the patch could receive nutrients from the host heart while releasing secretion including hepatocyte growth factor, vascular endothelial growth factor (VEGF), and insulin‐like growth factor 1 (IGF‐1) to further promote cellular and vascular regeneration in the injured area. In the MI rat model, after 3 weeks of MN‐CSCs treatment, Masson's trichrome staining showed a notable increase in viable myocardium tissue and infarct wall thickness accompanied by a significant decrease of scar tissues (Figure 7B–D). Detection of left ventricular ejection fractions (LVEFs) confirmed the improved cardiac function after treating with MN‐CSCs (Figure 7E,F). It is noted that the microneedles’ intervention required open‐heart surgery. This meant that there might be potential surgical risks and post‐operative side effects, therefore minimal invasive procedures with microneedles need to be developed in the future. However, there is no denying that this kind of nontoxic MN‐CSCs effectively enhanced vascular neovascularization in the damaged areas and reshaped cardiac functions, providing a novel strategy for MI patients’ treatments.

**FIGURE 8:**
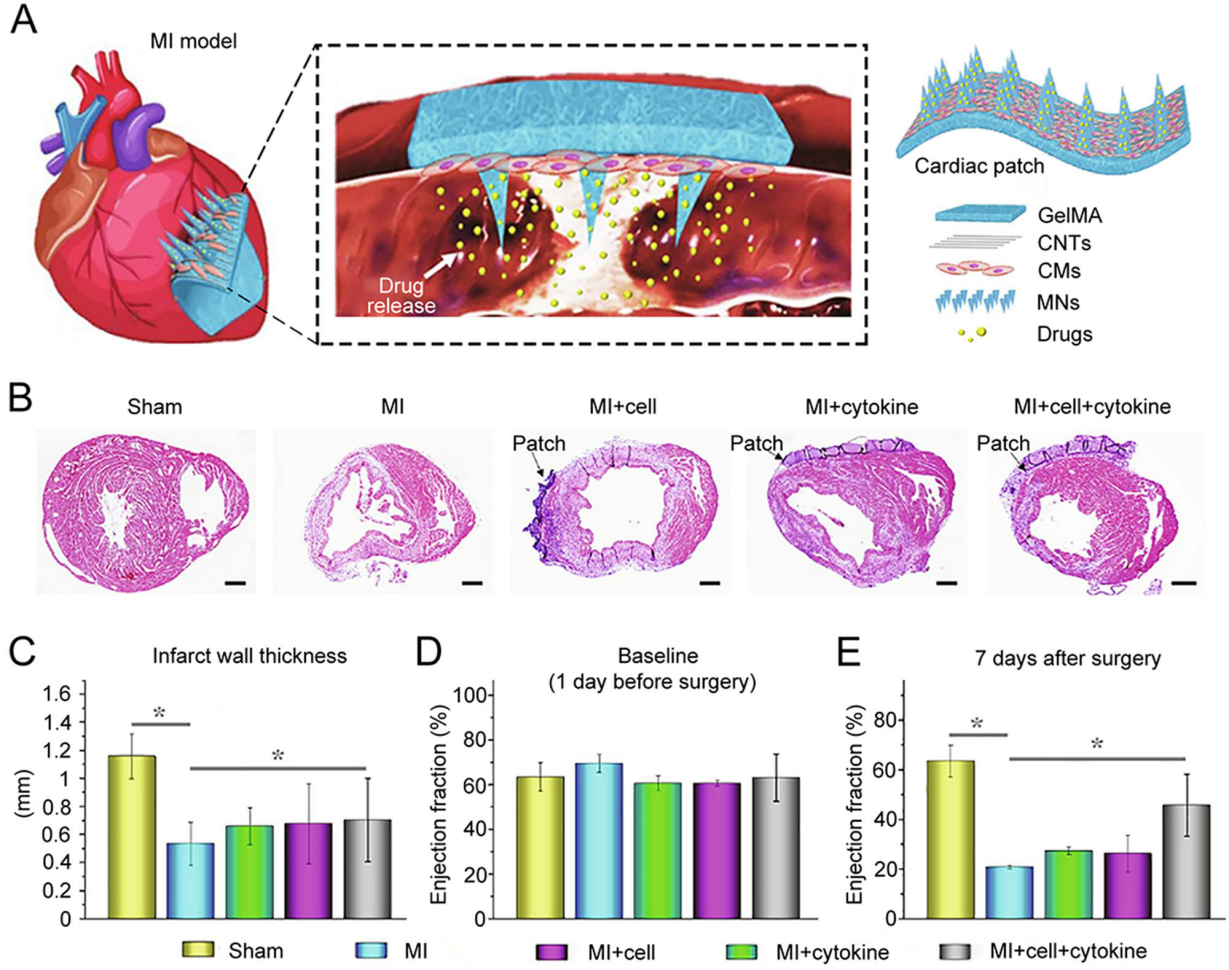
iPSCs‐derived CMs‐loaded conductive microneedles for the treatment of MI. (A) Schematic illustration of iPSCs‐derived CMs‐loaded CNTs‐integrated conductive MN array patch. (B) H&E staining results of sham group, MI group without treatment, MI+cell patch group, MI+cytokine patch group, and MI+cell+cytokine patch group, respectively (scale bar: 500 µm). (C) Quantitative analysis of left ventricular wall thickness. (D,E) LVEFs measured by echocardiography 1 day before surgery (baseline) and 7 days after surgery. ^*^ *p* < 0.05 when compared with MI group. Reproduced with permission.^[^ ^85^ ^]^ Copyright 2021, Elsevier

More recently, Zhao and his team designed conductive microneedles loaded with iPSCs‐derived CMs to treat MI (Figure 8A).^[^ ^85^ ^]^ iPSCs are obtained by inducing adult cells in body tissues such as skin and blood into cells resembling an embryonic state through reprogramming techniques.^[^ ^86^, ^87^ ^]^ iPSCs have been already available in clinically treating heart diseases.^[^ ^88^ ^]^ Carbon nanotubes (CNTs) can be a myocardial scaffold material due to their excellent electrical, photothermal, and mechanical properties,^[^ ^89^ ^]^ and they are proven to have the ability to induce CMs growth directionally.^[^ ^90^, ^91^ ^]^ In their study, parallel‐aligned CNTs were integrated with iPSCs‐derived CMs‐loaded microneedles using the multi‐layer fabricating strategy. The aligned conductive CNTs enhanced the information exchange between cells and ensured the directional differentiation of iPSCs into CMs before study in vivo. When applied, these directional distributed CMs in vitro were essential for heart disease treatment. To further enhance the treatment effect, VEGF and interleukin‐10 (IL‐10, an anti‐inflammatory cytokine) were encapsulated into needles. In the MI model experiment, normal cardiac morphology was evidenced from the images of H&E‐stained tissue sections from mice with a treatment of 7 days (Figure 8B). Moreover, the samples treated with cytokine‐encapsulated iPSCs‐microneedles showed the best thickening effect of the left ventricular wall (Figure 8C). The consequence of the LVEFs test confirmed the substantial recovery of cardiac functions (Figure 8D,E). Briefly, the CNTs‐integrated conductive microneedles ensured the oriented alignment of iPSCs‐derived CMs and synchronized activities. In addition, the versatile microneedles offer a broad prospect for iPSCs‐based therapy of cardiovascular diseases.

In addition to carrying stem cells, microneedles can also be used as adeno‐associated virus (AAV) carriers for the gene treatment of ischemic myocardial disease. AAV‐based gene therapy not only can mediate long‐term stable gene expression but also possess high safety, low immunogenicity, which receives increasing attention in the cardiac treatment field.^[^ ^92^, ^93^ ^]^ Moreover, the microneedle‐mediated injection method allows for the precise and controlled release of drugs at the lesion site. Based on these advantages, Shi et al. developed AAV‐loaded microneedles for ischemic heart disease treatment.^[^ ^94^ ^]^ Fluorescent labeling results verified the successful gene transfection with uniform and precise distribution of AAV at injected cardiac regions, which proved the feasibility of AAV‐VEGF‐loaded microneedles (MN‐AAV‐VEGF). According to Masson's staining and H&E staining results, MN‐AAV‐VEGF treated MI heart tissues had fewer infarcted areas and more distinct tissue remodeling. The use of VEGF promoted angiogenesis and fortified heart functions. It is worth noting that this AAV‐loaded microneedle patch can be applied through a small incision, thus reducing the infection risk caused by open‐heart surgery and increasing the therapeutic efficiency. Altogether, the clever combination of microneedles and AAV has in part addressed the challenge of how to deliver therapeutic genes to the injured heart and open the door to clinical application of gene therapy for heart diseases.

With the continuous upgrading of microneedle technology, microneedle implantation in the heart area can also be minimally invasive.^[^ ^22^ ^]^ In the study of Fan et al., the graphene oxide (GO)‐PVA microneedles only required a minimal opening of 4 mm for implantation and the special ability to self‐unfold could be activated under a NIR irradiation of 10 s. Additionally, the microneedles firmly adhered to the surface of heart even under strong extrusion force. VEGF was electrostatically loaded into the porous structure of microneedles through soaking. Considering the high drug loading efficiency and controlled release property of GO‐PVA microneedles, VEGF could sufficiently implement its function in the treatment of MI.

In summary, microneedles can be equipped with different stem cells, viruses, cytokines, and drugs to achieve targeting treatments of heart‐related diseases with minimal invasion. In addition, microneedles can be integrated with all sorts of organic or inorganic materials to reinforce their physical properties and therapeutic effects. In contrast to traditional methods of treating heart diseases, such as direct drug injections or open‐heart surgery, diverse microneedles bring people hope with novel cell or gene therapy.

### 3.5 Promoting neovascularization

Angiogenesis is the process of forming new blood vessels based on the development of existing capillaries from vascular bed.^[^ ^95^ ^]^ It involves a series of complex cellular activities and plays an important role in many physiological and pathological processes. Under normal physiological conditions, the angiogenesis process is tightly regulated by the dynamic balance between activation and inhibition of the related signaling factors. Nevertheless, under some abnormal conditions, inadequate neovascularization will lead to some unfortunate cases like cardiovascular and cerebrovascular diseases, slow wound healing, and insufficient tissue repair.

VEGF has been proved to be one of the proangiogenic molecules.^[^ ^96^ ^]^ Spontaneously, angiogenesis‐dependent diseases can be treated by enhancing the expression of VEGF. Liu et al. explored the AAV‐VEGF‐loaded GelMA microneedles to treat ischemic stroke.^[^ ^97^ ^]^ Microneedles were inserted into the ischemic core and penumbra of rats. The results showed uniform distribution and efficient transfection of AAV, which supported VEGF to regulate angiogenesis and improve neuronal survival rate. The microneedles enable local gene therapy through the precise delivery of AVV expressing human VEGF, exhibiting immense potential for other ischemic diseases.

Microneedles have progressively developed into an indispensable tool in the area of promoting vascular renewal. Wang's team proposed soluble microneedles with the synergistic effect of NO treatment and photothermal therapy in ischemic perforator flaps.^[^ ^98^ ^]^ Appropriate NIR irradiation with the well‐known abilities to inhibit inflammation, promote angiogenesis, and deposit collagen had already been applied in clinical wound healing.^[^ ^99^ ^]^ Additionally, NIR irradiation also played a critical role in the release process of NO gas from NO donor, facilitating subsequent neovascularization and reducing flap necrosis rate.

After vascular injuries happen, perivascular drug delivery is needed to reduce intimal hyperplasia, a vascular inflammatory response. A few years ago, Lee and co‐workers designed a paclitaxel‐loaded PLGA microneedle cuff for this purpose.^[^ ^100^ ^]^ The results of treating rabbit balloon injury model confirmed the superior drug delivery and therapeutic effect of this microneedle cuff. In the latest study, they developed a sirolimus‐loaded flexible silk fibroin microneedle wrap using the transfer molding method.^[^ ^101^ ^]^ Balloon‐injured rabbit abdominal aorta studies showed a significant reduction in neointimal formation. Microneedles permitted continuous delivery of anti‐proliferation drugs and substance exchange between blood vessels and extravascular tissues. Additionally, biocompatible PLGA and silk fibroin reduced the side effects of long term use. In short, microneedles have unparalleled advantages over other perivascular administration methods.

In conclusion, angiogenesis is a complex process regulated by multiple factors, and the occurrence of many diseases is associated with an insufficient number of blood vessels. Even though several strategies have been used to address this problem, in recent years an increasing number of microneedles have shown some promising results in the field of promoting neovascularization.

### 3.6 Accelerating wound healing

Wound healing is a complex and dynamic process, during which cells, tissue layers, and mediators interact in a highly sophisticated sequence.^[^ ^102^ ^]^ Many factors including dysregulated inflammation or hyperplasia of nonmigratory epithelial cells can lead to inadequate wound healing and further medical intervention is needed.^[^ ^103^, ^104^, ^105^ ^]^ Although traditional means such as sutures, staples, and medical glues are widely used in wound closure, they face side effects including scar formation and infection, poor adhesive, and uncontrollable polymerization.^[^ ^106^, ^107^, ^108^ ^]^ To solve these problems, microneedles are developed to inhibit infection and accelerate wound healing. With loading antimicrobial drugs, hemostatic drugs, and pro‐regenerative molecules, microneedles can realize anti‐bacteria, hemostasis, anti‐inflammation, and tissue regeneration, respectively.^[^ ^109^ ^]^ Thus, there is great potential for microneedles to be a safe, painless, and effective drug delivery system in the wound healing process.

Ensuring that microneedles do not come off is the first priority during the design of microneedles. As an illustration of adhesive technologies, Jeon et al. designed hydrogel‐forming double‐layered microneedles (DL‐MN) consisting of a swellable mussel adhesive protein (MAP) and non‐swellable silk fibroin (SF).^[^ ^110^ ^]^ MAP is biocompatible, biodegradable, and also has surface‐independent adhesion.^[^ ^111^ ^]^ SF is a natural fibrous protein with excellent biocompatibility and mechanical strength, as well as poor water solubility.^[^ ^112^ ^]^ In their study, SF was used as an in situ hard base to support effective tissue insertion and MAP/HA was used to interact with tissues via physical/chemical interactions to achieve trauma closure. In the rat model with ileum defect, DL‐MN could instantly close bleeding internal wounds, reduce the inflammatory response and inhibit the secretion of lymphoid follicles (LFs), a substance with potential harm in excess,^[^ ^113^ ^]^ at the gastrointestinal tract. Moreover, the rat model with full‐thickness skin incision was also established to confirm the healing effect of external wounds. DL‐MN was superior to the conventional suture treatment because of the better and faster therapeutic effect as well as the lower infection risk. To sum up, this adhesive DL‐MN could heal both external and internal wounds in wet and dynamic complex microenvironment. Of note, a similar MAP and SF‐based double‐layered adhesive microneedle was also applied for cardiac disease treatment, reliably demonstrating the versatility with promising efficacy.^[^ ^114^ ^]^

**FIGURE 9:**
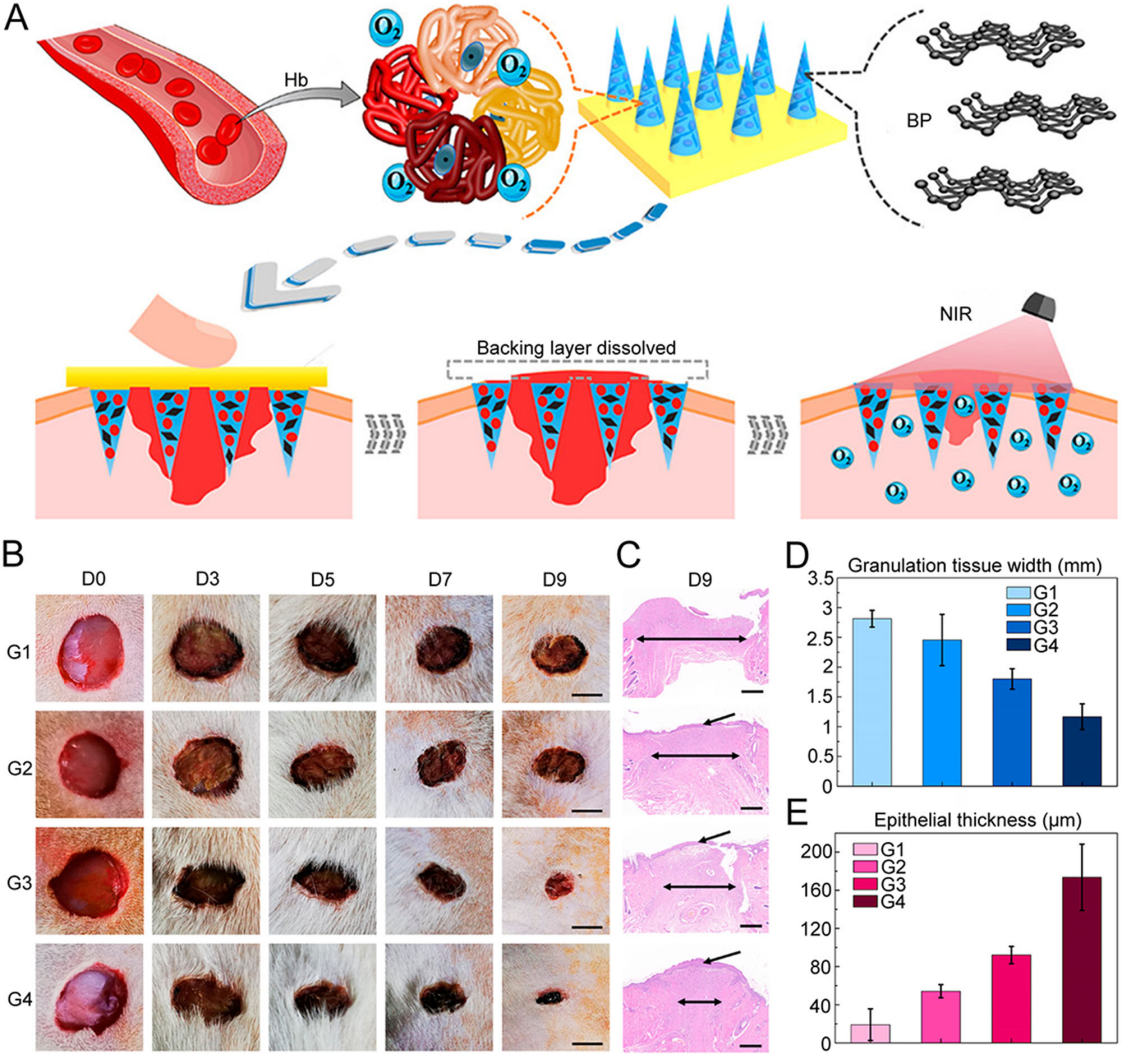
NIR‐responsive separable microneedles containing BP quantum dots and oxygen‐carrier Hb for promoting wound healing. (A) Design and working mechanism of the microneedle patch. (B) Images of the skin wounds of different groups on days 0, 3, 5, 7, and 9 (scale bar: 0.5 cm). (C) Corresponding H&E staining of the wound beds on day 9 (scale bar: 500 µm). Quantitative analysis of the (D) granulation tissue width and (E) epithelial thickness on day 9. G1: control group, G2: BP microneedles group, G3: BP + Hb microneedles group, G4: BP + Hb + NIR microneedles group. Reproduced with permission.^[^ ^117^ ^]^ Copyright 2020, American Chemical Society

In the wound healing process, adequate oxygen is a crucial factor promoting cell proliferation and tissue remodeling.^[^ ^115^ ^]^ Therefore, efforts have been made to produce various oxygen carriers to meet different conditions and requirements.^[^ ^116^ ^]^ Currently, black phosphorus (BP)‐loaded separable responsive microneedles with the ability to controllably deliver oxygen were designed for wound healing, as shown in Figure 9A.^[^ ^117^ ^]^ BP, a two‐dimensional material, is emerging in many fields due to its large NIR extinction coefficient, high photothermal conversion efficiency, easy fabrication process, and superior biocompatibility.^[^ ^118^ ^]^ Researchers used biocompatible GelMA as microneedle tips doped with a certain amount of BP quantum dots and oxygen‐carried hemoglobin (HB), and soluble PVA as the supporting base. The NIR‐responsive and controlled oxygen releasing microneedles were fabricated by the template replication method. After insertion, the base detached quickly owing to the dissolution of PVA, leaving GelMA microneedle tips inside the skin. When under NIR irradiation, BP nanoparticles achieved photothermal conversion to increase the local skin temperature and subsequently reduced hemoglobin's ability to bind oxygen, leading to a responsive oxygen release. Then the type I diabetic rat model with a wound at a diameter of 1 cm on the skin surface was established to assess the therapeutic effect of such microneedles (Figure 9B). Animal experiment results showed that microneedles with BP+Hb+NIR had the smallest granulation tissues and the thickest regenerated epithelial tissues, indicating the excellent wound healing ability of this patch (Figure 9C–E). The oxygen‐carried microneedles had a satisfactory curative effect, laying the groundwork for future application in the field of wound repair and other related biomedical areas.

Also using NIR light, biocompatible two‐dimensional transition metal carbides and nitrides (MXene)‐integrated adenosine microneedles were prepared using the template replication approach.^[^ ^119^ ^]^ Adenosine is an active component commonly found in the human body and it was shown that an increased concentration of adenosine at injury sites could promote the wound healing process.^[^ ^120^ ^]^ 3‐(acrylamido)phenylboronic acid‐integrated polyethylene glycol diacrylate hydrogel was utilized to construct microneedles. The presence of borate linked a dynamical covalent bonding for the controlled release of adenosine, providing a basis for subsequent wound treatment. The microneedles could be heated up under NIR irradiation due to the excellent photothermal conversion capability of MXene. This heating process was shown to be positively correlated with the concentration of MXene and NIR exposure time, which effectively accelerated the release of adenosine. To assess the practical therapeutic value of adenosine‐loaded MXene microneedles in vivo, they then constructed a trauma animal model for investigation. Adenosine‐loaded microneedles accelerated vascular regeneration and promoted wound repair compared to patches without adenosine. In particular, the group with adenosine and NIR irradiation showed the best therapeutic efficacy. These results suggested that the MXene‐integrated adenosine microneedles with photothermal effect had great potential in wound repair.

In addition to preparing NIR‐triggered microneedles using photothermal materials, pH‐sensitive coatings can be used to wrap microneedles in response to the pH changes in traumatic skin. As an illustration, a porous PLGA coating was added to a stainless steel‐based microneedle patch.^[^ ^23^ ^]^ After packing with gelatin porogenic agent, the desired drug was placed in the coating's pores and then these pores were blocked by a Eudragit S100 film. Eudragit S100 is a pH‐sensitive polymer that is soluble in interstitial fluid at alkaline pH conditions but insoluble in acidic microenvironments.^[^ ^121^ ^]^ In other words, in the alkalescent microenvironment of the wound area (pH 7.5), the protective film would be dissolved, pores were exposed and drugs inside were released for therapeutic use. However, at healthy skin pH (pH 4.5) levels, drug diffusion was not significant. Due to the excellent biocompatibility and easy manufacture of PLGA coatings, this pH‐sensitive platform can be used to carry and transport a variety of antibiotics as well, and it's available to regulate the number of carried drugs by controlling the ratio of porogenic agents to the polymers.

A chronic wound is a long‐lasting wound without complete repair. This has a serious impact on the quality of patients’ life and is now one of the most pressing challenges in the medical care field. For this reason, there is a growing need for chronic wound management. Much effort has been put into exploring a variety of wound treatment devices. Among them, microneedles can achieve responsive drug release in addition to minimally invasive and painless drug delivery, and the construction of microneedles with special structures or integrated electronic components provides a variety of functions beyond drug administration therapy. Particularly for chronic wounds, an incredibly positive impact on the wound recovery process would be exerted if the microneedles could be implemented with an intelligent data collection system and an electronic record of how the wound changes over time.^[^ ^122^ ^]^

**FIGURE 10:**
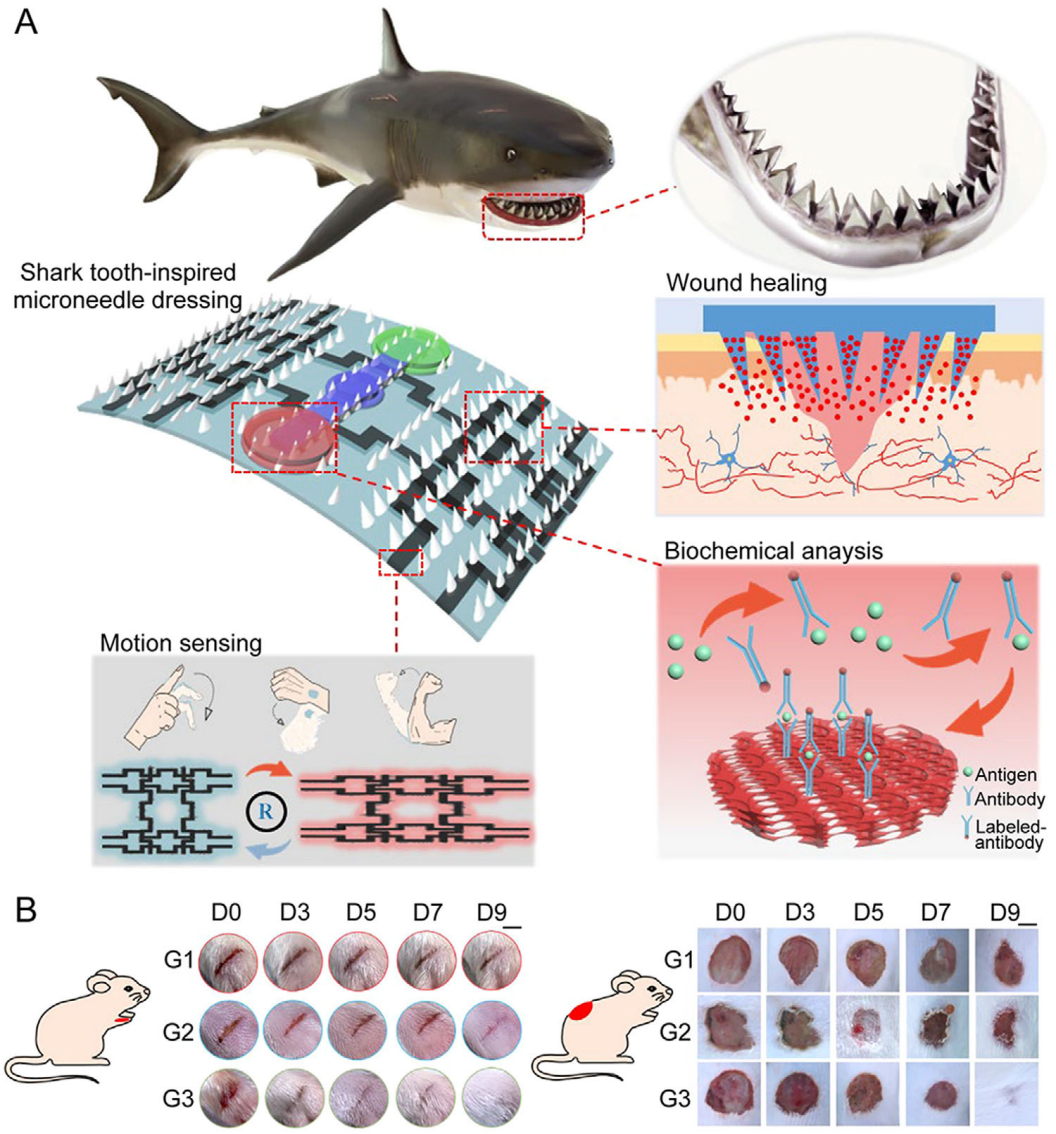
Shark tooth‐inspired multifunctional microneedles for intelligent chronic wound management. (A) Schematic illustration of the microneedle dressing for motion sensing, biochemical analysis, and wound healing. (B) Images of strip‐shaped wounds (scale bar: 0.5 cm) cut on a diabetic mouse's foot and circular wounds (scale bar: 1 cm) cut on a diabetic mouse's back under different treatments. G1: control group, G2: treated with blank microneedle dressing group, G3: treated with hEGF‐loaded microneedle dressing. Reproduced with permission.^[^ ^123^ ^]^ Copyright 2021, American Chemical Society

Recently, a smart wound management system based on microneedles was investigated by He's group.^[^ ^123^ ^]^ The microneedles that mimicked the bite of shark teeth were fabricated by stretching and tilting the laser‐engraved negative polydimethylsiloxane (PDMS) molds (Figure 10A). This bionic shape allowed to keep stable adhesion of microneedles over a prolonged period in the complex environment of the wound site. On a flexible polyurethane (PU) base, microfluidic channels were constructed using the origami technique, driving wound secretions, such as calprotectin, IL‐6, and CRP, to enter the inverse opal (IO) photonic crystal (PC) area via the capillary force. The PC‐enhanced fluorescence increased the sensitivity of wound secretions analysis.^[^ ^124^ ^]^ Simultaneously, a porous IO PC coating was applied on the silk fibroin‐based needle tips to enhance drug loading capacity, and a temperature‐responsive *N*‐isopropylacrylamide (NIPAM) hydrogel was used to achieve controlled drug release.^[^ ^123^ ^]^ To further advance the functions of the patch, Ti_3_C_2_T*_x_* MXene electro circuits were printed on the base to monitor patients’ movement and help reduce strenuous stretching. Diabetic mice were used to validate the chronic wound healing effect. Human epidermal growth factor (hEGF) was loaded onto the microneedles and animal experiments results showed that microneedles significantly improved the wound healing rate (Figure 10B), providing an all‐in‐one system for comprehensive and intelligent wound management. In another work of the same group, the authors prepared mosquito mouthparts‐inspired microneedles using NIPAM hydrogel for the construction of IO PC's three‐dimensional ordered structure to achieve personalized and programmable management of irregular wounds.^[^ ^125^ ^]^ With the emergence of wearable devices and developments in adhesion technology and antimicrobial strategy, the future holds great promise for multifunctional microneedles integrating diagnosis, treatment, and monitoring.

**FIGURE 11:**
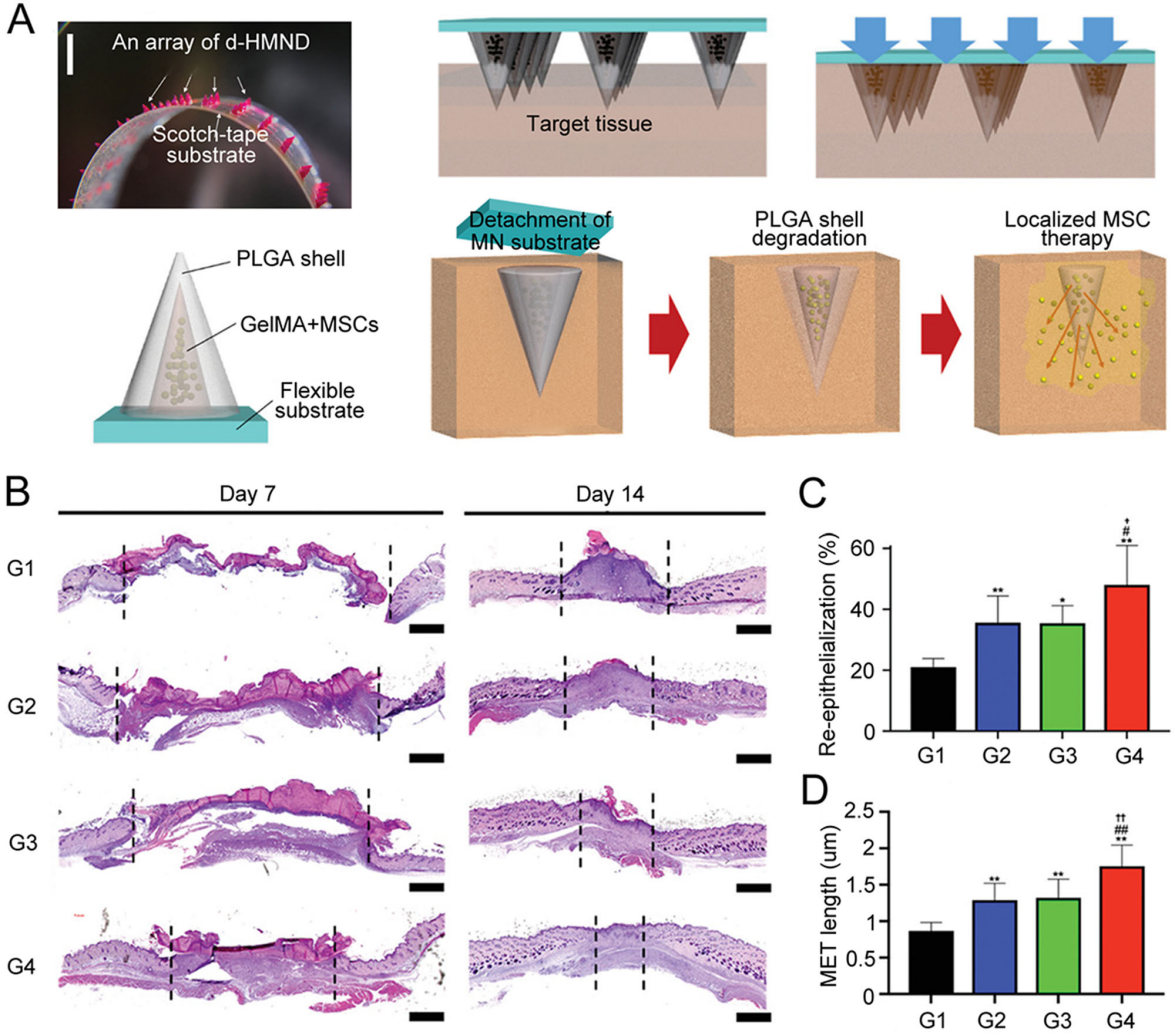
MSCs‐loaded detachable hybrid microneedle device for wound healing. (A) Design and working mechanism of d‐HMND (scale bar: 200 µm). (B) Histologic images of wound bed treated by each method on days 7 and 14 (scale bar:1 mm). G1: injury group, G2: MSCs injection group, G3: d‐HMND without MSCs group, G4: d‐HMND with MSCs group. (C) Re‐epithelialization and (D) MET length after 2 weeks. ^*^ *p* < 0.05 and ^**^ *p* < 0.01 compared with injury group. ^#^ *p* < 0.05 and ^##^ *p* < 0.01 compared with MSCs injection group; ^†^ *p* < 0.05 and ^††^ *p* < 0.01 compared with d‐HMND without MSCs group. Reproduced with permission.^[^ ^127^ ^]^ Copyright 2020, Wiley‐VCH

MSCs have broad healing potential. If MSCs are introduced into injured tissue, they can promote the formation of new blood vessels, reduce inflammation, and maintain cell viability.^[^ ^126^ ^]^ In clinical treatment, precise deposition of massive MSCs is required to ensure persistent cell functioning. To break through these existing bottlenecks, a minimally invasive approach using microneedles to deliver a bioactive depot of MSCs was developed by Khademhosseini's group.^[^ ^127^ ^]^ The researchers used a soft GelMA matrix to accommodate MSCs and sheltered the mixture with a stiffer PLGA capsule. Through microneedles, MSCs could gently enter the tissue and reach the target sites. Once delivered to the wound site, the PLGA shell degraded slowly. This slow degradation process restricted MSCs at the wound site with continuous release of therapeutic factors through the channels in the shell. For aesthetic reasons and to reduce possible contamination from external substances, a detachable hybrid microneedle depot (d‐HMND) was designed using the double‐sided scotch tape, as shown in Figure 11A. In the mouse wound model, compared to direct injection with an equivalent amount of MSCs into the wound and blank d‐HMND device without MSCs, d‐HMND containing MSCs significantly accelerated wound contraction according to the histologic images (Figure 11B). The evaluation of re‐epithelialization condition and migrating epidermal tongue (MET) length also confirmed that d‐HMND with MSCs treatment accelerated wound recovery rate (Figure 11C,D). Overall, in addition to being used as a treatment for skin wounds, the d‐HMND device can work as the vehicles of living stem cells and delivery them to various tissues and organs to realize the premium regeneration effect.

Wound healing is a dynamic and complex process and microneedles offer a superior method of wound treatment against direct injection of therapeutic drugs and surgical suturing. With the continuous evolution of manufacturing technologies and the advent of innovative ideas, a wide range of microneedles has become, or hold promise as effective tools in facilitating wound healing. Table 2 provides a summary of recent representative studies about the applications of microneedles for wound healing promotion. Microneedles can be applied to load a wide range of functional substances and advanced with diverse fabrication materials and methods, giving them a considerable potential for wound treatment and management.

**TABLE 2:** Summary of microneedles used in wound healing

Microneedles	Characteristics	In vitro/vivo models	Outcomes	Ref.
PVA matrix loaded with zeolitic‐imidazolate‐frameworks‐derived porphyrin‐like metal centers nanoparticles	808 nm NIR laser‐triggered Liquid band‐aid	In vitro: *S. aureus* In vivo: *S. aureus*‐infected mouse model	Disrupt bacterial membrane by local hyperthermia and peroxidase‐like activity of PMCS Antibacterial rate (92.9%) and wound healing ratio (86.6%) A mass of fibroblasts and blood vessels	^[^ ^182^ ^]^
Hollow microneedles fabricated by multimaterial FDM 3D printing with hard resin as needles and flexible polymeric resin as the base	Individualized bandage for chronic diabetic wounds Programmable drug delivery platform with peristaltic micropumps Integration of reusable electronics	In vitro: crust and necrotic tissue covering the viable tissue In vivo: full‐thickness diabetic wound model	Increase healthy cells’ drug bioavailability and average wound closure rate (95%) New hair growth Reduce MMP9 (an enzyme involved in the extracellular matrix degradation) expression	^[^ ^183^ ^]^
NHGs embedded in porous PEGDA‐microneedles (NHGs: *N*‐diazeniumdiolates‐loaded copper‐benzene‐1,3,5‐tricarboxylate metal‐organic framework (MOF) is encapsulated with graphene oxide)	NO gas: facilitate angiogenesis and vasodilation Photothermally responsive release of NO Deeper and more precise release of NO Reduction of toxicity of copper ions through 4‐MAP modified‐MOF	In vitro compatibility test: NIH/3T3 cells In vivo: type I diabetic rat model	Excellent compatibility of porous NHG‐microneedles Reduction in wound area (NHG‐MN+NIR group: reduce to 1.0 ± 0.3%) Promotion of re‐epithelialization (NHG‐MN+NIR group: granulation tissue thickness reached 1.39 ± 0.02 mm)	^[^ ^184^ ^]^
Magnesium organic frameworks (Mg‐MOFs) mixed with poly(γ‐glutamic acid) (γ‐PGA) hydrogel as needles with γ‐PGA hydrogel and graphene oxide‐silver nanocomposites mixture as the base	Multifunctional platform including anti‐inflammation and antibacterial effect, promote angiogenesis and tissue repair	In vitro antibacterial test: *S. aureus*, *E. coli*, and *P. aeruginosa* In vitro cell migration test: Human umbilical vein endothelial cells In vivo: living diabetic mice with round cutaneous wounds on the back	73% inhibition rate of DPPH free radicals at a Mg‐MOF concentration of 60 µg mL^–1^ Significant decrease in wound area and granulation tissue width 73% inhibition rate of DPPH free radicals at a Mg‐MOF concentration of 60 µg mL^–1^	^[^ ^185^ ^]^
Zeolitic imidazolate frameworks‐8 (ZIF‐8) encapsulated photo‐crosslinked methacrylated hyaluronic acid (MeHA) based hydrogel microneedles	Zn‐MOF: nanoporous materials with anti‐bacterial property MeHA: biodegradable and ductile	In vitro antibacterial test: *E. coli* and *S. aureus* In vivo: full‐thickness infected cutaneous defect rat model	Maximum granulation tissue thickness (1.93 ± 0.06 mm) Significant decrease in IL‐6 expression Massive collagen deposition	^[^ ^186^ ^]^
Polydopamine‐gelatin mixture as base and PEGDA‐sodium alginate as tips with polymyxin loaded both	Suction‐cup‐structured concave chambers surrounded Excellent wet/dry adhesion; polymyxin Broad‐spectrum antimicrobial property Self‐healing ability	In vitro antibacterial test: *E. coli* In vivo model: knee osteoarthritis rat model	Excellent flexibility and adhesion ability (withstand weights in excess of 240 times microneedles’ mass) High *E. coli* killing rate Significant recovery of joint lesions after glucocorticoid‐loaded microneedles treatment	^[^ ^187^ ^]^
Chitosan microneedles loaded with VEGF encapsulated poly(N‐isopropylacrylamide) (pNIPAM) hydrogel	Chitosan: porous structure, excellent antimicrobial, and wound healing properties pNIPAM hydrogel: temperature‐responsive Smart drug release	In vitro antibacterial test: *E. coli* and *S. aureus* In vivo: severely infected wound model	Bacterial mortality up to 99% Enhanced airflow between internal and external environments Optimal regeneration levels brought by increased temperature induced by inflammatory reaction	^[^ ^188^ ^]^

### 3.7 Fulfilling hair regrowth

Hair loss due to a variety of causes is a common phenomenon in the human body. Scientists have been working hard to find the real reason for hair loss and help solve the related problems plaguing people. Studies have shown that hair growth is relevant to the growth of hair follicles (HFs) and shows a periodicity divided into anagen (growth phase), catagen (regression phase), and telogen (dormancy phase),^[^ ^128^ ^]^ which is largely regulated by hair follicle stem cells (HFSCs).^[^ ^129^ ^]^ Normally, HFSCs are in a quiescent state, but they can be activated to start a new hair growth cycle if needed. However, if HFSCs cannot be activated effectively, the cyclic development of HFs is broken. This means the growth phase is shortened while the dormancy phase is prolonged, with the macroscopic manifestation of hair loss.

**FIGURE 12:**
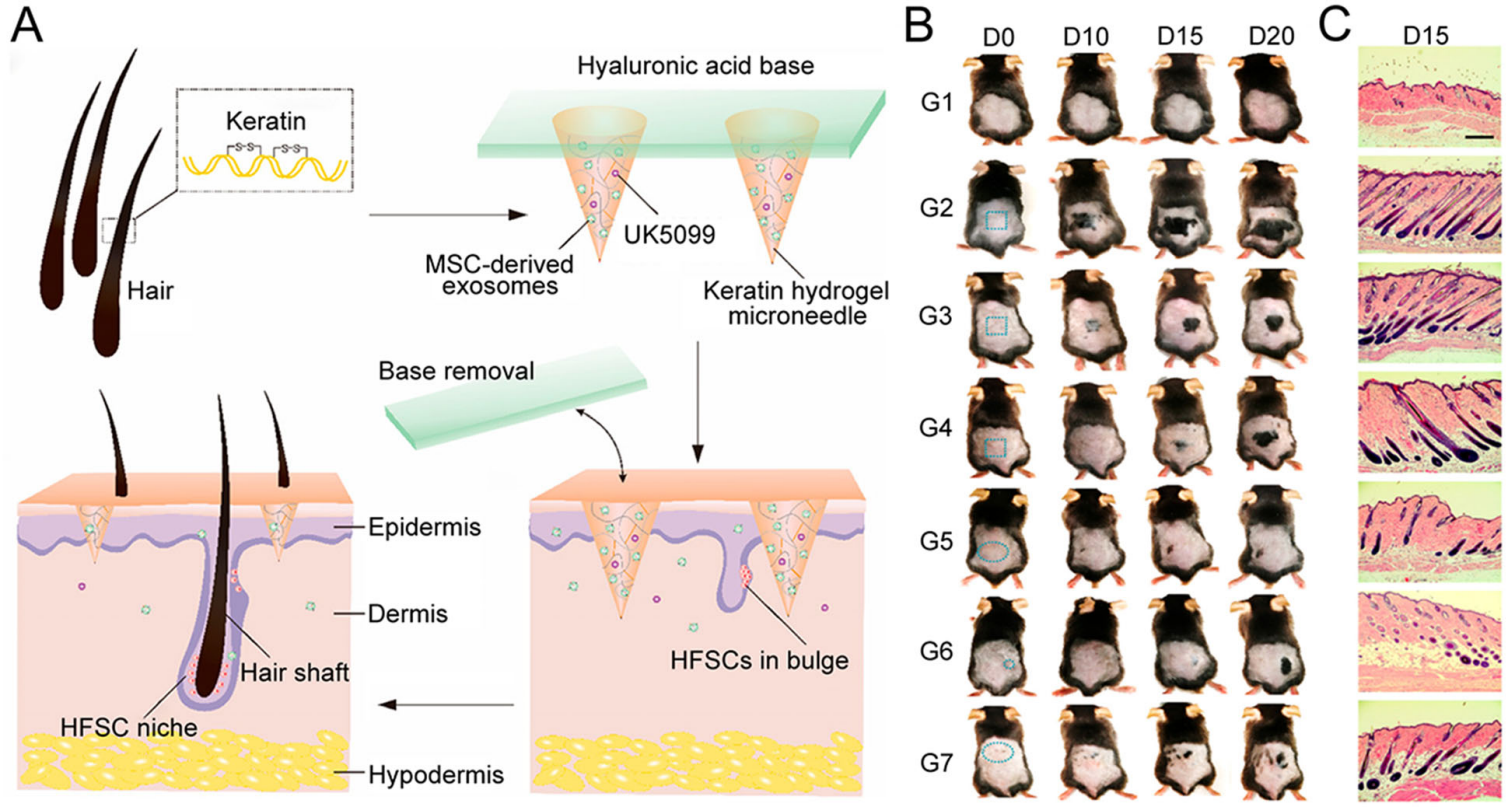
Detachable microneedles made from keratin for hair regrowth. (A) Schematic illustration of hair loss therapy through a detachable microneedle patch system. (B) Photographs of mice treated with different conditions. G1: control group, G2: exosomes and UK5099‐loaded HMN, G3: UK5099‐loaded HMN, G4: exosomes‐loaded HMN, G5: topical UK5099, G6: subcutaneous injection of exosomes, G7: topical minoxidil. (C) H&E staining of mice skin at day 15 postadministration (scale bar: 300 µm). Reproduced with permission.^[^ ^132^ ^]^ Copyright 2019, American Chemical Society

In the study of the metabolic process and regulatory mechanism of HFSCs, researchers identified the EXOs^[^ ^130^ ^]^ and a small molecule drug UK5099^[^ ^131^ ^]^ that could activate HFSCs and induce a new hair growth cycle. However, traditional delivery methods for these two substances have low transdermal efficiency, short drug residence time, and require frequent injections. Given this, Gu's team designed microneedles to deliver EXOs and UK5099 simultaneously.^[^ ^132^ ^]^ The microneedles were made of biocompatible and biodegradable keratin hydrogels with a network structure based on intermolecular disulfide bonds, which gave the microneedles high mechanical strength and sustained drug release.^[^ ^133^, ^134^ ^]^ The HA patch base could be easily detached owing to the rapid absorption of tissue fluids following insertion. After removing the HA base, the remaining needle tips could act as the drug reservoirs in skin and sustainably release the embedded contents, which enabled the transdermal delivery of activators to the HFSCs niche and accelerated hair regeneration (Figure 12A). The researchers confirmed the effectiveness of the microneedles in promoting hair growth through animal pharmacodynamic experiments (Figure 12B,C). As a result, the microneedle‐treated group showed significant hair regrowth, which offered a more efficient hair loss treatment compared to subcutaneous injection of EXOs and topical application of UK5099 and minoxidil. Notably, the patch can be easily adjusted in shape and size according to users' specific situations, exhibiting enormous potential to achieve personalized treatment.

Similarly, Hong and the co‐workers prepared HA‐based microneedles containing keratin, melanin nanoparticles, and EXOs from human amniotic MSCs.^[^ ^135^ ^]^ Low‐color‐temperature yellow light (1900 K light) was used to synergistically promote hair regrowth in the mouse model. The authors’ idea was to imitate the process of germination and growth of seeds in nature. EXOs are used to regulate the microenvironment around HFs, like the soil in which seeds grow. 1900 K light is like sunlight rejuvenating HFs to enter the growth phase. This co‐lighting approach provides a new way for hair regrowth and has the opportunity to be applied in the improvement of other skin‐related conditions.

Although current clinical treatments for hair loss primarily rely on follicular transplantation and drug injection,^[^ ^136^, ^137^ ^]^ more and more studies have shown the premium benefits of microneedles as alternative treatment means.^[^ ^138^ ^]^ Androgenetic alopecia (AGA) is one type of popular hair loss disease, but effective treatments are lacking. A few years ago, Lahiji et al. developed carboxymethyl cellulose‐based soluble microneedles to deliver valproic acid (VPA) for the treatment of AGA.^[^ ^139^ ^]^ The microneedles not only enabled precise VPA medication, but also created micro‐incisions up‐regulating the Wnt/β‐catenin signaling pathway in the HFs growth cycle. In light of that hair growth could be promoted by the re‐epithelialization following trauma,^[^ ^140^ ^]^ this study tactfully applied micro‐invasions coming from microneedles to accomplish a similar or even better curative effect. Not only did it reduce pain, but it could also be used with medication, providing a latent optimization for future AGA treatments.

**FIGURE 13:**
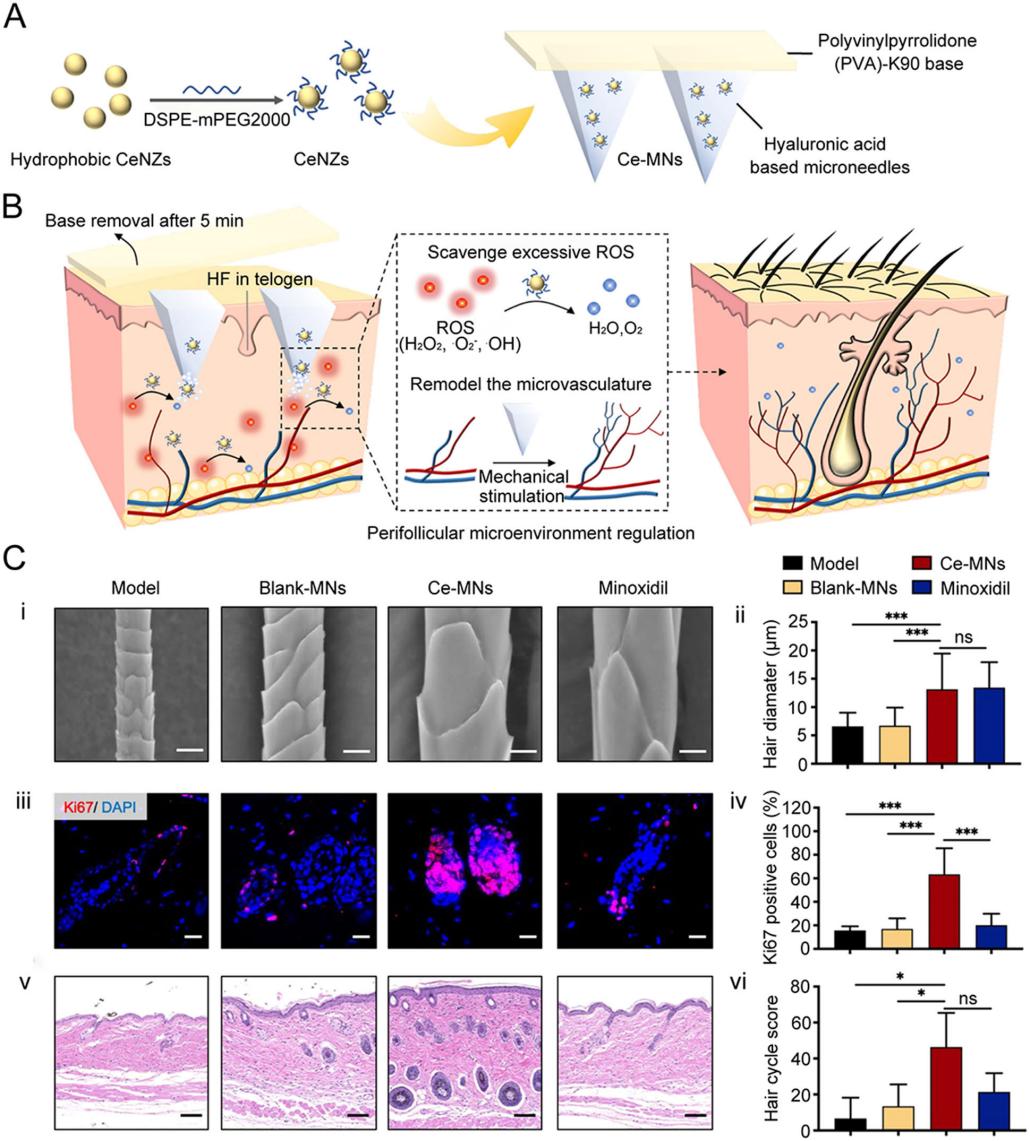
CeNZs‐loaded microneedles for scavenging ROS in AGA treatment. (A) Fabrication of CeMNs. DSPE‐mPEG_2000_ is used to transform hydrophobic CeNZs to hydrophilic CeNZs. (B) Working mechanisms of CeMNs to promote hair regrowth. (C) Evaluation of Ce‐MNst hair regeneration effect. (i) SEM images of regenerated hair at day 28 postdepilation (scale bar: 10 µm). (ii) Diameter of regenerated hair at day 28 postdepilation. (iii) Expression of Ki67 (red) and DAPI (blue) after 10 days (scale bar: 20 µm). (iv) Quantitative analysis of Ki67 positive cells at day 10 postdepilation. (v) H&E staining of the treated skin at day 10 postdepilation (scale bar: 100 µm). (vi) Hair cycle score calculations at day 10 postdepilation. ns: *p* > 0.05, ^*^ *p* < 0.05, ^***^ *p* < 0.001. Reproduced with permission.^[^ ^143^ ^]^ Copyright 2021, American Chemical Society

With further research on AGA, people found that high‐concentration of ROS and inadequate vascularization in the perifollicular microenvironment could lead to a dysfunction of HFs niche, thereby resulting in AGA.^[^ ^141^, ^142^ ^]^ Recently, ceria nanozymes (CeNZs)‐loaded microneedles (Ce‐MNs) were designed to cure AGA.^[^ ^143^ ^]^ Ce‐MNs consisted of HA‐based needles encapsulating DSPE‐mPEG2000‐modified CeNZs and removable PVP‐K90 base (Figure 13A). CeNZs in the patch could scavenge excessive ROS and reshape the oxidative microenvironment around HFs. At the same time, the mechanical stimulation (200–300 µm deep into the skin) caused by the microneedles could promote the generation of microvasculature around HFs. The two‐pronged approach promoted hair regeneration in AGA patients through a series of reactions (Figure 13B). The authors established the AGA mouse model to evaluate the hair regeneration effect using Ce‐MNs. Compared to the topical administration with minoxidil, a first‐line drug used for treating AGA, the regenerated hairs induced by Ce‐MNs exhibited a healthier state with more appreciable diameter, more proliferation around HFs, more augmented HFs in the anagen phase, and earlier conversion of hair phase transition (Figure 13C). In summary, these novel Ce‐MNs composed of soluble needles and a detachable base could scavenge ROS and promote angiogenesis to remodel the microenvironment around HFs for treating hair loss. For people suffering from long‐term AGA, the clinical translation or practical applications of these microneedles are expected, which have the enormous potential to revitalize hair.

Microneedles are already known to be an alternative to invasive or time‐consuming regimens in some cases. So far, increasing advantages have been proved for microneedles in fulfilling hair regrowth, such as simple operation and painless, foreshadowing the potential of microneedles in hair loss treatments.

## 4 CONCLUSION AND FUTURE PERSPECTIVE

Among the main methods currently established to repair tissue and organ damage caused by trauma or diseases, organ transplantation is greatly limited by donor difficulties and ethical issues. Artificial organs, although promising, are difficult to be used on a large clinical scale because their morphologies and functions are far weaker or less than those of real organs. Innovative therapeutic approaches promoting endogenous repair and regeneration of tissues or organs may solve medical challenges and issues such as rejection of organ transplants, thereby meeting a significant need for clinical treatment.

The processes of self‐repair and regeneration of tissues or organs require the involvement of a variety of cells, extracellular matrices, and regulatory factors. In the field of tissue engineering and regenerative medicine, microneedles are often used as scaffolds to support cell adhesion and growth through interacting with cells to induce their migration, proliferation, and differentiation. Besides, microneedles can regulate the microenvironment of injury sites, such as releasing inflammation suppressing drugs, delivering signaling factors and growth factors. Intelligent microneedles can be used to achieve precise treatments in response to external/internal stimuli. Most notably, the presence of microneedles has the potential to make some cutting‐edge therapies such as stem cell therapy and gene therapy a reality. Although using microneedles in tissue engineering and regenerative medicine is still in its infancy, exciting breakthroughs can be expected in the future. The following lists the prospects of microneedles and the practical problems accompanied.

1. The processes of self‐repair and regeneration of tissues or organs require the involvement of a variety of cells, extracellular matrices, and regulatory factors. In the field of tissue engineering and regenerative medicine, microneedles are often used as scaffolds to support cell adhesion and growth through interacting with cells to induce their migration, proliferation, and differentiation. Besides, microneedles can regulate the microenvironment of injury sites, such as releasing inflammation suppressing drugs, delivering signaling factors and growth factors. Intelligent microneedles can be used to achieve precise treatments in response to external/internal stimuli. Most notably, the presence of microneedles has the potential to make some cutting‐edge therapies such as stem cell therapy and gene therapy a reality. Although using microneedles in tissue engineering and regenerative medicine is still in its infancy, exciting breakthroughs can be expected in the future. The following lists the prospects of microneedles and the practical problems accompanied.
2. To meet more complex medical needs, in addition to excellent mechanical properties and biocompatibility, endowing microneedles with new features like the responsive or controlled release of drugs should also be on the agenda. Meanwhile, it's also necessary to develop multifunctional microneedle systems like diagnosis and treatment integration.
3. Microneedles have been already reported in the repair of various tissues, such as tendon, skeletal muscle, periodontal tissue, bone, heart, skin, and hair. However, globally, there is a significant demand for the post‐damage regeneration of other vital tissues and organs such as the liver, kidney, and lung. Thus, it is urgent to conduct microneedle‐relevant research in these areas.
4. The transition from laboratory to clinic requires comprehensive and standardized cellular, animal, and clinical tests to demonstrate safety, reproducibility, and efficacy. It is also essential to fully understand the pathophysiology and individual medical needs to further improve the relevance and convenience of microneedles.

Overall, with the continuous improvement of basic and clinical studies, kinds of versatile microneedles will be developed and play important roles in the field of tissue engineering and regenerative medicine.

## CONFLICT OF INTEREST

The authors declare no conflict of interest.
